# Estimating the Axial Compression Capacity of Concrete-Filled Double-Skin Tubular Columns with Metallic and Non-Metallic Composite Materials

**DOI:** 10.3390/ma15103567

**Published:** 2022-05-16

**Authors:** Pavithra Chandramouli, Revathy Jayaseelan, Gajalakshmi Pandulu, Veerappan Sathish Kumar, Gunasekaran Murali, Nikolai Ivanovich Vatin

**Affiliations:** 1 Department of Civil Engineering, B.S. Abdur Rahman Crescent Institute of Science & Technology, Chennai 600048, India; cpavitra89@gmail.com (P.C.); gajalakshmi@crescent.education (G.P.); 2 Faculty of Civil Engineering, Architecture and Geodesy, University of Split, 21000 Split, Croatia; 3 Peter the Great St. Petersburg Polytechnic University, 195251 St. Petersburg, Russia; murali_22984@yahoo.com (G.M.); vatin@mail.ru (N.I.V.)

**Keywords:** ACC, CFDST, ANN, steel tubular, concrete-filled tubes, axial compression capacity, artificial neural network

## Abstract

This research focuses on estimating the ACC (axial compression capacity) of concrete-filled double-skin tubular (CFDST) columns. The study utilised algorithms and ‘six’ evaluation methods (XGBoost, AdaBoost, Lasso, Ridge, Random Forest Regressor and artificial neural network (ANN) architecture-based regression) to study the empirical formulae and utilise the parameters as the research’s features, in order to find the best model that has higher and accurate reliability by using the RMSE and R^2^ scores as performance evaluation metrics. Thus, by identifying the best model in empirical formulae for estimating the ACC of CFDST, the research offers a reliable model for future research. Through findings, it was found that, out of the existing evaluation metrics, the ABR for AFRP, GFRP and Steel; RFR for CFRP; and RR for PETFRP were found to be the best models in the CFDST columns.

## 1. Introduction

**Figure 1:**
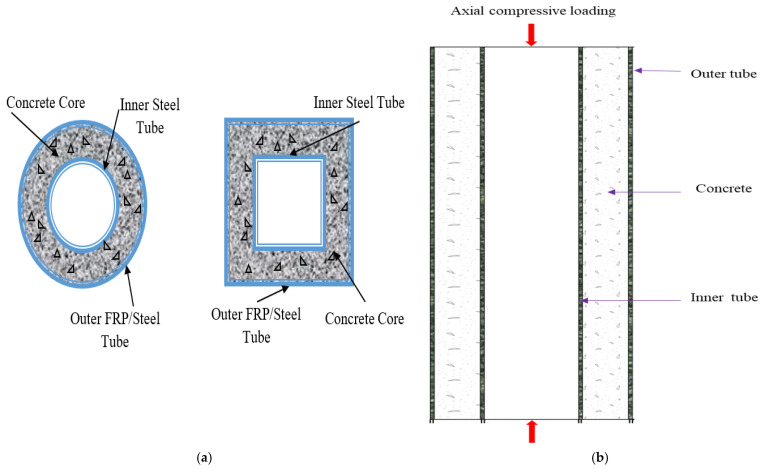
(**a**) Typical CFDST columns (Circular and Square: Cross-sectional) and (**b**) Compressive loading of CFDST.

In the construction industry, the most widely adopted materials and structures are the “double-skin tubular columns” that are filled with concrete and made with steel. Generally, the CFDST columns have two layers (outer and inner skin) of concrete-filled tubes, unlike the normal single-skin tubular columns as indicated in Figure 1. In CFDST, the steel acts as the base for the tubular columns and has offered the construction industry better outcomes in terms of core strength (axial compression), tensile strength, ductility, load-bearing capacity [1], structural strength, yield strength, toughness [2], heavy vehicle load-capacity, elastoplastic capacity [3], heavy-wind, wearing and erosion, etc. [4], mainly in the countries such as China, USA, Japan and India [5]. The concept of “double skin” composite construction was first devised for use in submerged tube tunnels [6]. The construction industry more widely adopts double-skin tubes than single-skin-based concrete-filled steel tubular (CFST) columns since they offer more tensile strength and load [7].

Later, the CFST column was altered to the concrete-filled fibre tube column (CFFT), which aimed to boost structural durability, attracted attention towards the non-metallic confinement in addition to the undisputed corrosion resistance of shells made of polymer pipes reinforced with glass fibre, basalt or carbon fibre [8]. CFDST columns with both outer and inner steel tubes provide several advantages, including enhanced section modulus, strong seismic and fire performance, better global stability, favourable building ability, reduced weight, use of inner tube space if needed and good damping properties [9]. It is also possible to employ Fibre-Reinforced Polymer (FRP) material as a replacement for steel in construction due to its anti-corrosive characteristics, as steel is an energy-intensive material. In addition, as compared to the manufacture of steel, resin and fibre have a low environmental effect. The steel–concrete–steel was modified to a novel form of FRP–concrete–steel, and the failure behaviour of the specimen was due to the initial Poisson’s ratio of concrete being smaller than that of steel; the steel tube moves radially faster than the concrete, so the two components remain in contact. After the elastic range, the concrete begins to deform faster and separates from the steel tube and develops plasticity as a consequence; the outer layer tends to provide the confinement and improve the axial load capacity.

This study focuses on analysing outer tubes made of steel and FRP and inner steel tubes. The FRP materials such as Aramid (AFRP), Basalt (BFRP), Carbon (CFRP), Glass (GFRP) and Poly-Ethylene Terephthalate (PETFRP) have been utilised in construction due to their features, including high elasticity, cost-effectiveness, reduced shrinkages, and environmental and mechanical damages [10,11].

Though there are several other studies on the ACC [12,13], steel tubular columns [9,14], hence concrete-filled double skin steel tubular (CFDST) columns, have several benefits over concrete-filled steel tubular (CFST) columns. Among them are: (1) a stronger fire resistance even without a fire protection layer on the exterior steel tube, (2) a higher fire resistance even without a fire protection layer on the outer steel tube, (3) a higher retention of their load-bearing capability, preventing concrete spalling, and removing the need for reinforcement, (5) an increase the strength, ductility, and energy absorption of the formwork, and (6) a decrease in the weight of the formwork at the base of the column [2,9,14,15,16,17]; studies on DSTC with FRP have revealed that this composite system, which consists of a steel tube within the FRP tube with concrete sandwiched in between, combines the benefits of all three components to make a high-performance structural component. The versatility of DSTC cross-sectional designs allows them to meet unique structural and architectural requirements. The FRP tube and the steel tube efficiently contained DSTCs, resulting in an extremely ductile behaviour [18,19,20,21,22]. A DSTC hollow section column was chosen to create a lightweight construction. From an environmental perspective, the use of FRP results in a more sustainable environment by lowering the column’s inherent energy levels. Power cables, telephone lines and drainage pipes may all be accommodated in the inner tube’s hollow.

In the last few decades, considerable exercises have been made to employ smart computing algorithms to solve civil engineering issues. Vast research was carried out in the field of artificial intelligence. The sub-category of artificial intelligence is Machine Learning (ML), which it performs through algorithms and facilitates by enriching the previous datasets/experience. With a nominal input and the eradication of human errors, the soft computing algorithms would instinctively study and enhance itself over time. The beams and columns are designed and tested through machine-language-based algorithms, and estimations of the capacity are calculated through machine learning evaluations. The beams’ and columns’ failures offer the researchers better accuracy and precision that could provide better ACC in the construction. Similarly, the ‘loss rate’ or ‘percentage error’ in the machine language also offers the research developers better algorithms for estimating the ACC and preventing accidents and huge disasters [23].

There are various CFDST types, such as circular-square, square-circular, square, circular, hexagonal-circular, rectangular, hexagonal, octagonal and round-ended rectangular [24,25,26], with varied materials such as FRP and Steel [27]. This study, however, concentrates upon concrete-filled tubes with a double skin. To estimate the ACC through ANN in the CFDST columns, the research focuses on the general parameters of the CFDST columns and adopts the parameters as the variable factors for the research development. Thus, the study focuses on CFDST in general without a specific structure and hence the evaluation and estimation techniques in research have been broadened. Henceforth, the standard empirical formulae’s parameters will estimate the axial compression capacity in the CFDST columns.

Through the successes and failures of the existing CFDST column-based models, the research aims at utilising the empirical formulae’s parameters as variables in this research model towards estimating the ACC through the ANN. the ability to estimate the ACC through ANN-R in concrete fills is unknown, and thus the need for the research is eminent and justifiable. Thus, the research will analyse and evaluate the ACC through ANN-R towards measuring the CFDST columns through statistical evaluation methods such as “regression”, where Ridge Regressor, Lasso Regressor, Random Forest Regressor, AdaBoost Regressor and XGBoost Regressor will be adopted along with ANN-R based optimisations. Hence, the research will contribute to future evaluators with base knowledge and facts about the FRPs and steel tubular columns with double-skin tubes and concrete infills in the tubes. The computational models developed by the authors Le et al. (2021) [28] investigated the Rectangular CFST. Lee et al. (2014) [29] investigated the NN application towards predicting concrete and mortar in cement properties. Otieno et al. studied the level of corrosion in RCFST. Le and Phan (2020) [28] examined the prediction of ACC for RCFST through a hybrid machine learning ML model. Hou and Zhou (2022) [30] predicted ACC in C-CFST through ML method. The models used regression techniques, NN and ML methods for predicting ACC. Mai et al. (2022) [31] developed the ACC prediction model for SCFST columns. They analysed and concluded that ML models are predicted and reliable to the experimental results and utilising hybrid models or on-or-more regression results in higher accuracy in ACC prediction.

The other objective of the current study is to develop opensource, Python-based ML models to estimate the axial load-carrying capacity of CFDST columns to further help researchers utilise the developed models to improve the framework once additional experimental data are available [32].

### Pre-Existing Formulae and Design Codes

There are several techniques and methods for estimating the ACC load capacity in civil engineering research. In this research, the utilisation of six categorisations of statistical calculations (i.e., existing formulae) for estimating the steel-based concrete-filled tubes with double skin steel tubes is carried out. For instance, the ACI equation in estimating the ultimate axial strength (*P_u_* in kN) of CFDST is the most standard formula in the statistical method. Researchers could alter and modify the variables accordingly based on the material composite and strength. The ACI equation and the first CFDST equation developed by Uenaka et al. [33] resembles our equation, since the researchers adopted the equation and superimposed the steel tube’s strength through their formula. The formulae are as follows:

ACI Code:

(1) $${\left(P_{u}\right)}_{ACI}=f_{syo}A_{so}+0.85f_{c}A_{c}+f_{syi}A_{si}$$

Uenaka et al. [33]:

(2) $$P_{u}=f_{syo}A_{so}+f_{c}A_{c}+f_{syi}A_{si},$$

where $f_{syo}$ denotes the outer tube’s yield strength, and $A_{so}$ so represents the outer tube’s cross-sectional area. Similarly, $f_{syi}$ denotes the inner steel tube’s yield strength, and $A_{si}$ represents the inner tube’s cross-sectional area. The concrete annulus cross-sectional area is denoted by $f_{c}$, and the compressive strength is represented by $A_{c}$.

## 2. Statistical Analysis

By adopting the parameters, i.e., the inner and outer tube’s yield strengths and the cross-sectional areas of the inner and outer tubes, the study estimates the ACC in CFDST columns through statistical evaluation techniques. The following are the adopted evaluation techniques:

- Random Forest Regression (RFR): The RFR method in machine-learning-based algorithms is a process/analysis where regression, classification and other functions that work upon a multitude decision tree during the training phase of datasets that finally offers the outcomes as “classes” aka “classification” or prediction value of average/mean values (regression) that represents the individual trees.
- XGBoost Regression (XGBR): This is an approach that is utilised by researchers for supervised regression-based models that work upon the decision tree approach. In general, the XGBoost is utilised in various ranges of solving problems and applications such as regression, classification, and prediction-based user-defined and ranking problems.
- AdaBoost Regression (ABR): ABR is an estimation process (meta-estimator) where initially it fits the regressor upon the original dataset, and later it additionally fits regressor copies to the same dataset by adjusting the instance weights based on the prediction’s recent error estimation.
- Lasso Regression (LR): LR is a linear regression in ML that utilises shrinkage (i.e., the process of shrinking data values directed to central point/mean value). It generally aims at minimising the prediction error towards quantitative variables, unlike other regression techniques, and makes use of model parameters through constraints which results in “zero” shrink or with a lesser shrink value.
- Ridge Regression (RR): The ridge approach is generally adopted by researchers towards “multicollinearity” problems. This approach/model tunes the data that has suffered from multicollinearity damage/error, where unbiased least-squares and large variance-based predictions are found far away from the original mean values.
- ANN Regression (ANN-R): In regression, the Artificial Neural Network generally predicts the input functions through the output variable (example: binary) and functions as a classifier (class). To predict and estimate complicated problems in machine language and machine-learning-based algorithms, ANN architecture is widely utilised by researchers along with optimisers (Optimisation: Adaptive Moment as Adam and Gradient Algorithm as RMSprop).

This research makes use of six categorisations of statistical calculations (formulae) for estimating the steel-based concrete-filled tubes with a double skin of steel tubes. The formulae are:

### 2.1. Random Forest Regression (RFR) through Gini Gain

(3) $$Gini=1-\sum_{j}p_{j}^{2},$$

where *p* is the sample proportion belonging to the node.

### 2.2. XGBoost Regression (XGBR)

(4) $$f\left(x\right)\approx f\left(a\right)+f^{\prime }\left(a\right)\left(x-a\right)+\frac{1}{2}f^{\prime \prime }\left(a\right){\left(x-a\right)}^{2}\;{ℒ}^{\left(t\right)}≃\sum_{i=1}^{n}\left[l\left(y_{i,}{\hat{y}}^{\left(t-1\right)}\right)+g_{i}f_{t}\left(x_{i}\right)+\frac{1}{2}h_{i}f_{t}^{2}\left(x_{i}\right)\right]+\Omega \left(f_{t}\right),$$

where ℒ denotes CART learners’ function, a represents predicted value, *xC*-a denotes new learner of *t*, *x* denotes the objective function of the Taylor theorem, *f(x)* represents the loss function, and y represents the actual label.

### 2.3. AdaBoost Regression (ABR)

(5) $$a_{t}=\frac{1}{2}ln\frac{\left(1-TotalError\right)}{TotalError},$$

where $a_{t}$ denotes alpha value, and total error represents total misclassifications in training dataset.

### 2.4. Lasso Regression (LR)

(6) $$\overset{M}{\underset{i=1}{\sum }}{\left(y_{i}-{\hat{y}}_{i}\right)}^{2}=\overset{M}{\underset{i=1}{\sum }}{\left(y_{i}-\overset{p}{\underset{j=0}{\sum }}w_{j}\times x_{ij}\right)}^{2}+\lambda \overset{p}{\underset{j=0}{\sum }}\left|w_{j}\right|$$

### 2.5. Ridge Regression (RR)

(7) $$Cost\left(W\right)=RSS\left(W\right)+\lambda \times \left(sum\;of\;squares\;of\;weights\right)\;=\sum_{i=1}^{N}{\left\{y_{i}-\sum_{j=0}^{M}w_{j}x_{ij}\right\}}^{2}+\lambda \sum_{j=0}^{M}w_{j}^{2},$$

where in Equations (6) and (7), λ is the tuning parameter, *x* denotes the independent variable, *y* represents the dependent variable, and *w* denotes weight.

### 2.6. ANN Regression (ANN-R)

(8) $$x_{i,j}^{l}=\sum_{m}\sum_{n}w_{m,n}^{l}o_{i+m,j+n}^{l-1}+b_{i,j}^{l}$$

$$o_{i,j}^{l}=f\left(x_{i,j}^{l}\right)$$

$$\delta_{i,j}^{l}=\frac{\partial E}{\partial x_{i,j}^{l}}$$

$$\frac{\partial E}{\partial x_{i^{\prime },j^{\prime }}^{l}}=\sum_{m=0}^{k_{1-1}}\sum_{n=0}^{k_{2-1}}\delta_{i^{\prime }-m,j^{\prime }-n}^{l+1}w_{m,n}^{l+1}f^{\prime }\left(x_{i^{\prime },j^{\prime }}^{l}\right)$$

$$\frac{\partial E}{\partial w_{m^{\prime },n^{\prime }}^{l}}=\sum_{i=0}^{H-k_{1}}\sum_{j=0}^{W-k_{2}}\delta_{i,j}^{l}o_{i+m^{\prime },j+n^{\prime }}^{l-1}$$

## 3. Methodology for Analysis of ACC

**Figure 2:**
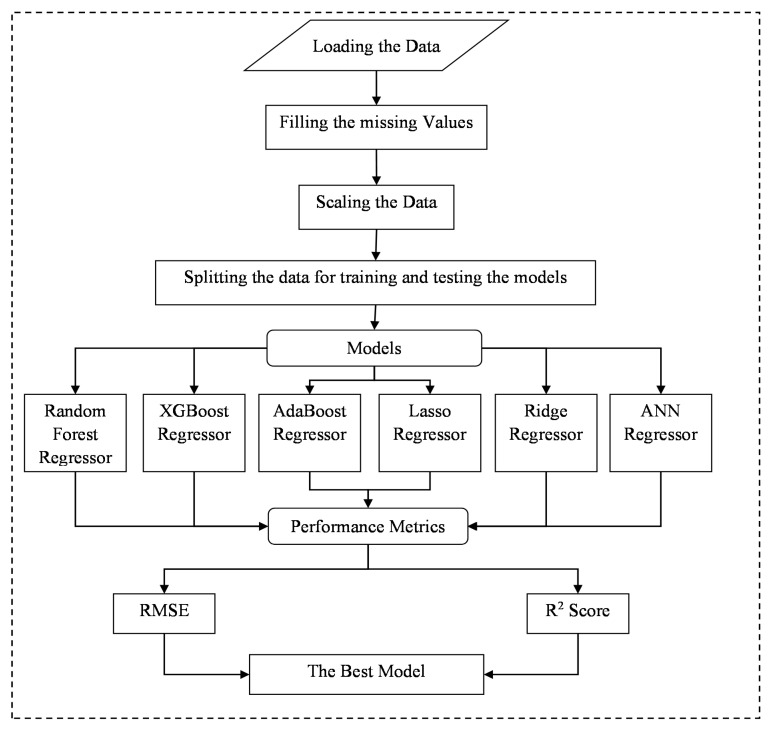
System Flow.

This research primarily focuses upon the estimation and the evaluation of ACC through the CFDST column. To estimate and evaluate the existing CFDST-based ACC values by utilising the empirical formulae’s parameters as the features/variables, the current study aims at identifying and developing a more advanced and new model that future research could adopt. Figure 2 represents the system flow for the adopted study.

## 4. Data Collection

The data for the research was gathered from 244 specimens of CFDST columns of AFRP, CFRP, GFRP, PETFRP and Steel given in Table A1 (refer to Appendix A). Each specimen has been observed and measured manually in this research for better understanding and accurate results. This type of data accumulation is considered to be “semi-experimental” design-based research.

The proposed dataset acquisition consists of processes such as data gathering, importing datasets, cleansing, pre-processing and, finally, selecting the better model. Once the datasets are cleansed and processed, the evaluation is carried out and the outcomes are obtained. Finally, the processed outcomes are compared for better model recognition in CFDST column-based research.

## 5. ANN Architecture, Parameters and Datasets

**Figure 3:**
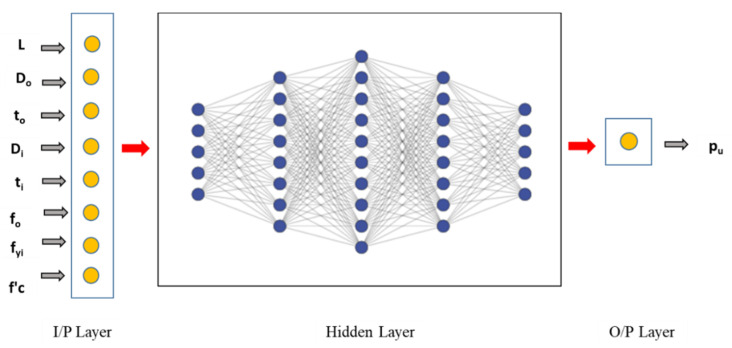
Schematic architecture ANN Model.

The schematic architecture for predecting the ACC with 8 input layers, multi hidden layers and one output layer is shown in Figure 3.

### 5.1. Input Parameters

The input parameters for the research in AFRP, CFRP, GFRP PETFRP and steel datasets total eight parameters each [*L* (mm), *D_o_* (mm), *t_o_* (mm), *D_i_* (mm), *t_s_* (mm), *f_o_* (Mpa), *f_yi_* (Mpa), f′_c_ (Mpa) and one output parameter *Pu* (kN)] with 38 specimens in AFRP, 59 specimens in CFRP, 61 specimens in GFRP, 22 specimens in PETFRP and 125 specimens in steel for the estimation of ACC in CFDST columns, where ‘o’ represents the outer skin and ‘*i*’ represents inner skin to determine the diameter (*D*), length (*L*), thickness (*t*), ultimate strength (*f_o_*), Yield strength (*fy*), Concrete’s Compressive strength (*fc*), and *P_u_* (kN), i.e., the Ultimate-Axial strength. The above-stated parameters are the most basic parameters in the CFDST columns towards estimation of ACC.

### 5.2. Network Architecture:

**Figure 4:**
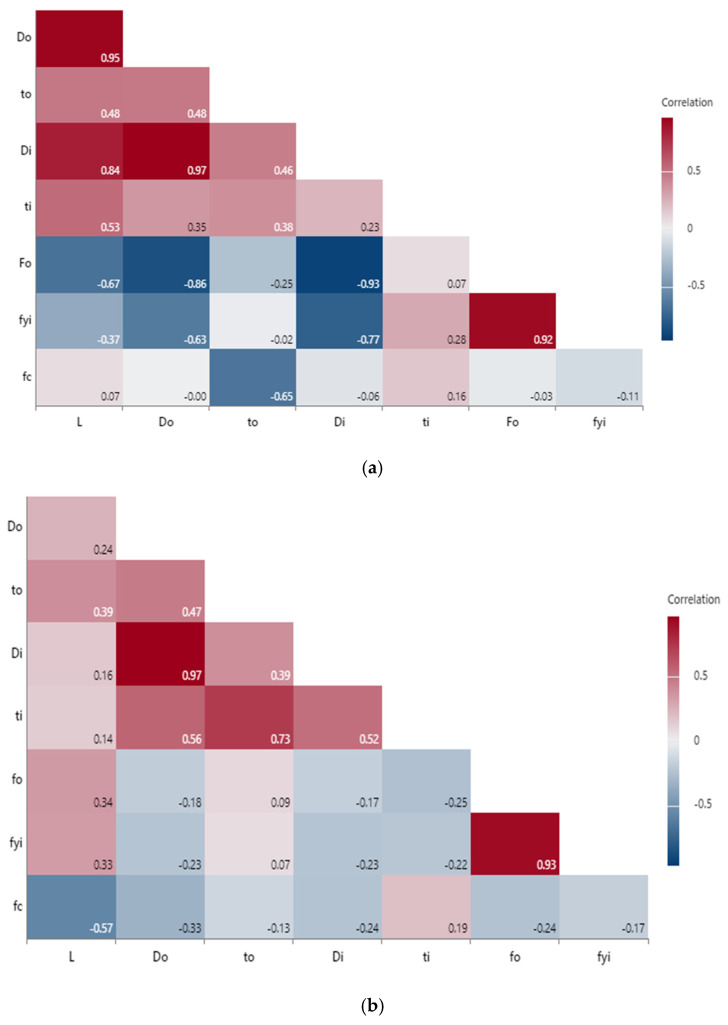
(**a**) Correlation matrix heat map of features of outer FRP tube and inner steel tube. (**b**) Correlation matrix heat map of features of outer and inner steel tubes.

The correlation matrix heat map of features with an outer FRP and inner steel tube are shown in Figure 4a. The correlation between the diameter of steel and the FRP tube, as well as the yield strength of the steel tube, and the ultimate strength of the FRP tube, as well as the diameter of the outer FRP and the length of the specimen, are represented by the coefficients 0.97, 0.95 and 0.92.

Similarly, the correlation matrix heat map of features with both outer and inner steel tube is shown in Figure 4b. The correlation between the diameters of both the inner and outer steel tube, as well as the ultimate strength and yield strength of both the inner and outer steel tubes, are represented by the coefficients 0.97 and 0.93.

**Figure 5:**
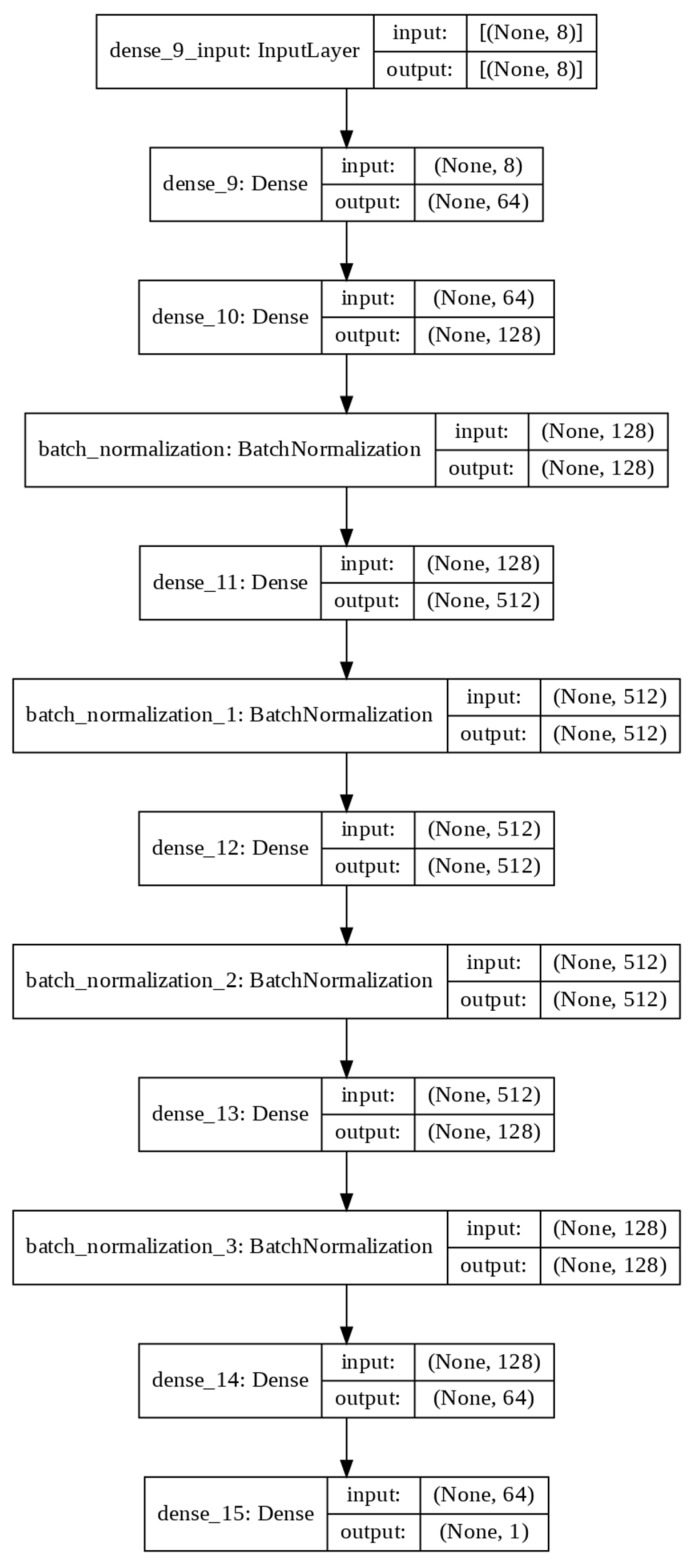
ANN Architecture.

A total of 244 specimens CFDST columns are utilised as inputs in the estimation process. The ANN architecture for each dataset is developed through ANN-hidden layers, where the datasets are trained and tested towards reliability, accuracy, validity and precision. Figure 5 represents the ANN architectures for the CFDST columns.

The architecture includes an 8 × 8 input layer as the first stage, followed by 2 dense layers with 8 × 64 bit, and 64 × 128 bit as the second stage. The following stages (3, 4 and 5) include 4 × batch normalisation layers (128 bit, 512 bit, 512 bit and 128 bit) followed by 4 × dense layers (128 × 512 bit, 512 × 512 bit, 512 × 128 bit and 128 × 64 bit), consecutively. In stage 6, an additional dense layer is added with a 64 × 1 bit. Thus, the ANN is layered for the CFDST column prediction model.

#### Auto-Encoder

The auto-encoder model (encoder–decoder) for the developed research is segregated into three stages. Firstly, data is processed through the “pre-processing” step, where the data is loaded, analysed and examined for missing values and filled in. Once the missing data are filled and pre-processed, the processed data is then passed onto ‘scaling’. Once the data are scaled, they are split into datasets with two labels: testing and training.

Secondly, the datasets are trained with the regression models (XGBoost, Adaboost, Random-Forest, Lasso, Ridge and ANN) through the respective coding algorithms in python. Once the sample data are trained, the remaining inputs are tested for validation of the models. The use of the Adam optimiser and RMSprop optimiser in this stage minimises the data loss. The outcomes (RMSE and R^2^ scores) are obtained and evaluated through the metric ‘evaluation technique’.

Finally, the outcomes are compared for evaluating the best model for estimating the ACC of CFDST columns.

The RMSE scores and R^2^ scores are obtained as estimated values/outcomes in the testing phase of the datasets, and they are compared again with the original/estimated outcome. If the results are similar and accurate, the same algorithm is applied to the remaining datasets of AFRP, CFRP, GFRP, PETFRP and STEEL through the testing process. The best empirical approach is evaluated through the obtained scores.

### 5.3. Pre-Processing Datasets

The pre-processing phase of the CFDST datasets contains two optimisations where ‘Adam’ and ‘RMSprop’ are utilised. The errors/losses are estimated through training and validation, and the outcomes are compared with ANN-R-based optimisations to obtain a better outcome. Initially, inputs (minimal datasets) are tested with the regressor models, and the outcomes are compared for reliability, and, once the results are satisfying, the same process is applied to the original data to compare the estimated outcomes of the datasets. The RMSE and R^2^ scores of each regressor model are the variables (performance metrics) for comparing the best outcomes of the ‘regression’ techniques.

## 6. Results and Findings

Through the evaluation techniques and formulae, the following outcomes were attained for the adopted regressors for the CFDST-based ACC predictions.

### 6.1. AFRP Dataset

**Figure 6:**
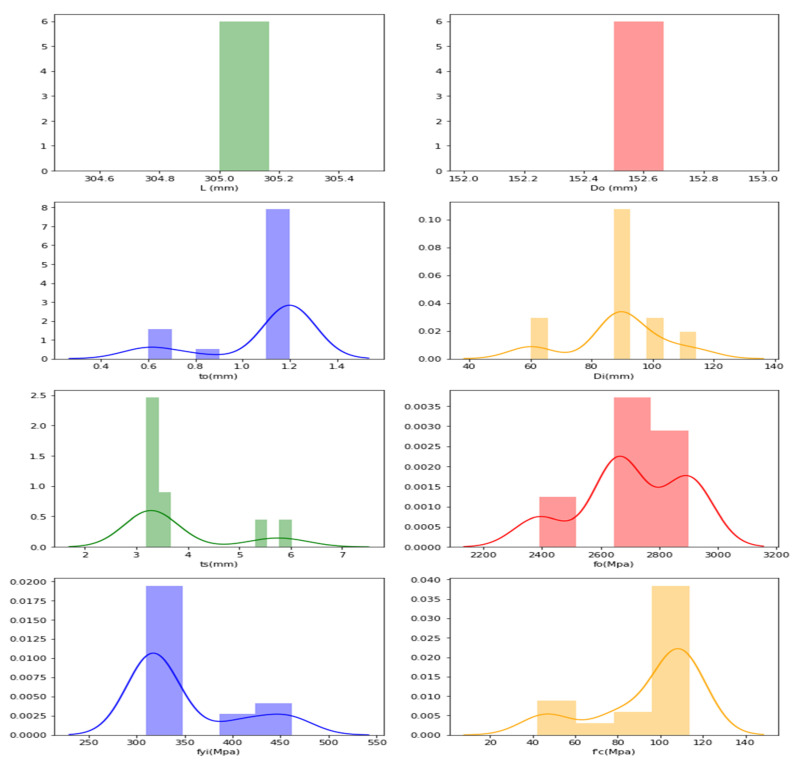
Frequency distribution in CFDST columns of AFRP representing features.

The frequency distributions of the features are plotted with the aim of verifying their distributions, and the following plots are obtained through graphical representation, as presented in Figure 6 for AFRP datasets.

The ‘*y*-axis’ in the graphs represents the frequency distributions’ ‘frequency’, and the obtained curve denotes that, as the data increases, the frequency distribution is achieved by joining the middle-points of the highest frequencies.

For the AFRP frequency distribution, it is understood that the data are distributed and found to have high ranges for *L* (300–310 mm), *D_o_* (150–160 mm), *t_o_* (1.1–1.2 mm), *D_i_* (90–95 mm), *t_s_* (3–4 mm), *f_o_* (2600–2900 Mpa), *f_yi_* (300–350 Mpa) and f′_c_ (80–110 Mpa).

Predictions through regressor techniques:

1. RFR: The estimated RMSE value is 537.12, where the R^2^ score is 0.58;
2. XGBR: The estimated RMSE is 542.54, where the R^2^ score is 0.70;
3. ABR: The estimated RMSE value is 510.00 where R^2^ score is 0.62;
4. LR: The estimated RMSE value is 660.82, where the R^2^ score is 0.37;
5. RR: The estimated RMSE value is 654.41, where the R^2^ score is 0.38.

Among the five regressor techniques above, ABR can be inferred as a good fit for AFRP in CFDST columns.

**Figure 7:**
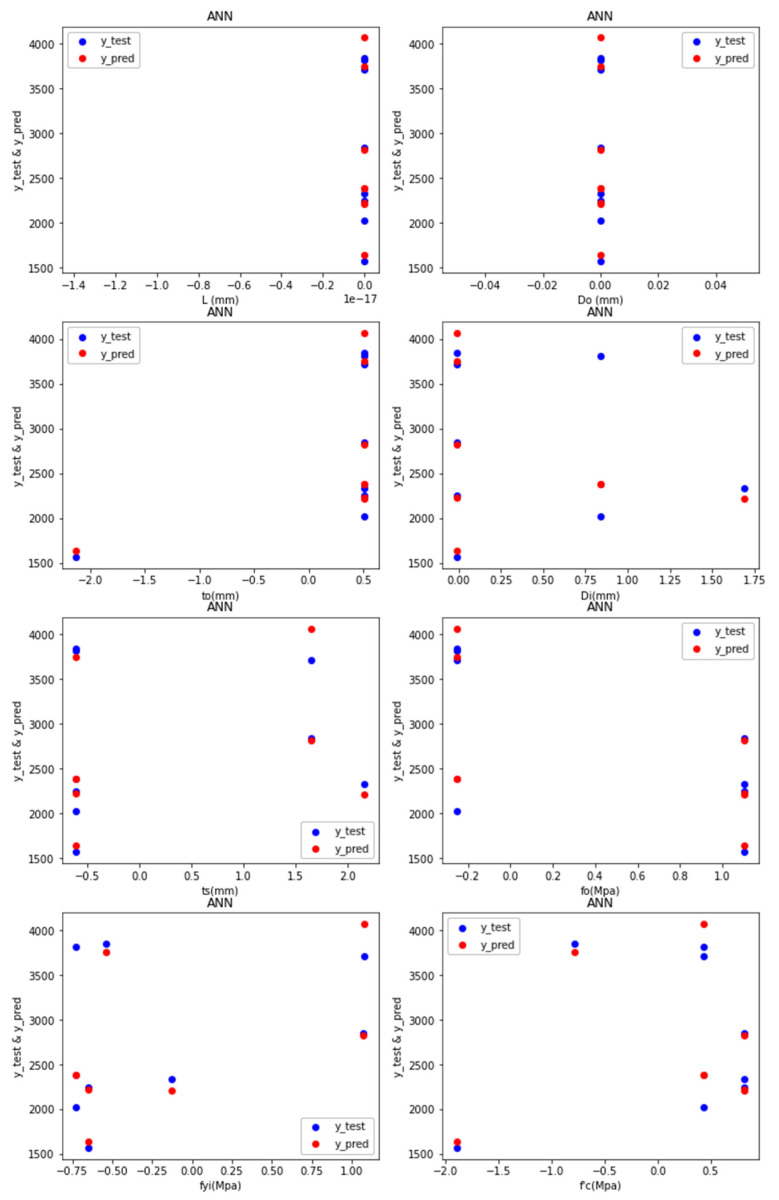
Predictions of ANN-R in AFRP.

1. ANN-R:

Predictions: Figure 7 depicts the test values and predicted values of the ANN-R in AFRP, with the blue dots representing the predicted values and the red dots representing the test values:

The predicted values (blue dots) and the test values (red dots) from Figure 7 are nearer to each other, denoting that the ANN-R in the AFRP model is a good fit.

**Figure 8:**
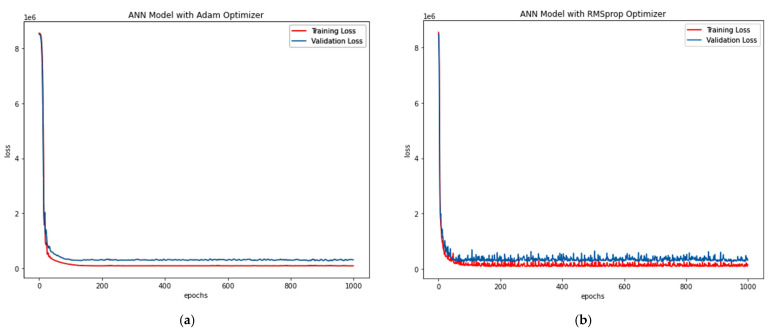
(**a**) Losses vs. Epochs of ANN-R Adam Optimisation in AFRP; (**b**) Losses vs. Epochs of ANN-R RMSprop Optimisation in AFRP.A.

- Adam Optimiser in ANN-R: The estimated RMSE value is 547.77, where the R^2^ score is 0.57, and the *P_u_* (kN) values of the predicted loss with respect to the values and original values are plotted in Figure 8a.
- RMS prop Optimiser in ANN-R: The estimated RMSE value is 558.12, where the R^2^ score is 0.55. The *P_u_* (kN) values of the predicted loss with respect to the original values are plotted in Figure 8b.

The losses vs. epochs in machine learning assist the researchers in training their developed ANN models. The epoch in ANN represents the training dataset’s full cycle with additional epochs and timings to validate how the model is trained. Loss is an attempt by the researcher to minimise the errors during the model’s training with ‘scalar value’. Thus Loss versus Epoch functions as an estimation that provides every data point towards measuring quantitative loss for a given epoch. Here, the loss versus epoch for ANN-R (AO) and ANN-R (RMSprop) optimisation is plotted, and the outcome is compared to determine the best optimiser.

From the above plots (Figure 8a,b) of ANN-R optimisation, it can be inferred that the ANN-R with an Adam optimiser performs better than the ANN regressor with a RMSprop optimiser.

### 6.2. CFRP Dataset

**Figure 9:**
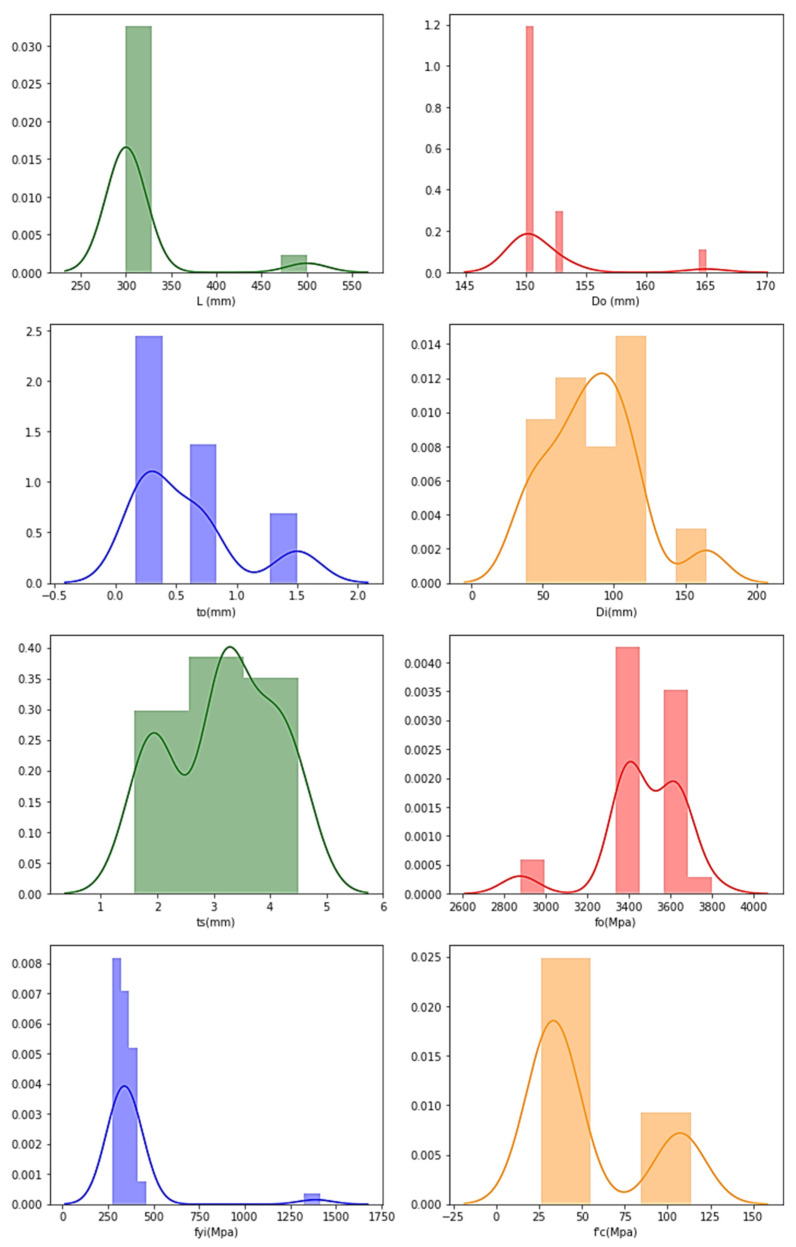
Frequency distribution in CFDST columns of CFRP-representing features.

The frequency distributions of the features are plotted with the aim of verifying their distributions, and the plots are obtained through graphical representation as shown in Figure 9 for the CFRP datasets.

Predictions through regressor techniques:

(a) RFR: The estimated RMSE value is 355.50, where the R^2^ score is 0.27;
(b) XGBR: The estimated RMSE is 355.22, where the R^2^ score is 0.27;
(c) ABR: The estimated RMSE value is 371.04, where the R^2^ score is 0.20;
(d) LR: The estimated RMSE value is 380.38, where the R^2^ score is 0.16;
(e) RR: The estimated RMSE value is 380.71, where the R^2^ score is 0.16.

Among the five regressor techniques above, RFR could be inferred as a good fit for CFRP in the CFDST columns.

**Figure 10:**
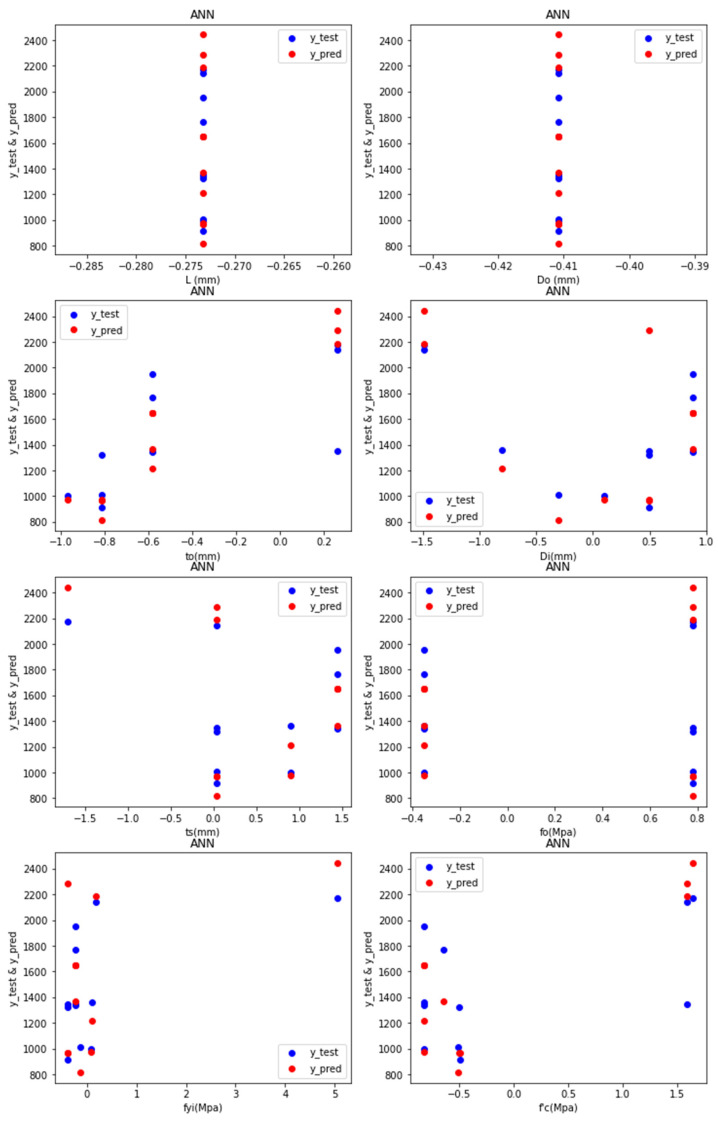
Predictions of ANN-R in CFRP.

(a) ANN-R:

Predictions: Figure 10 depicts the test values and predicted values of the ANN-R in the CFRP, with the blue dots representing the predicted values and the red dots representing the test values:

For the CFRP frequency distribution, it is understood from Figure 9 that the data are distributed evenly and the density of the frequency decreases as the data increases. The frequency distribution is high for *L_o_* (300–350 mm), *D_o_* (150–155 mm), *t_o_* (0.5–1.5 mm), *D_i_* (50–120 mm), *t_s_* (1.5–4.5 mm), *f_o_* (3200–3800 Mpa), *f_yi_* (250–500 Mpa) and f′_c_ (25–60 Mpa and 80–110 Mpa).

**Figure 11:**
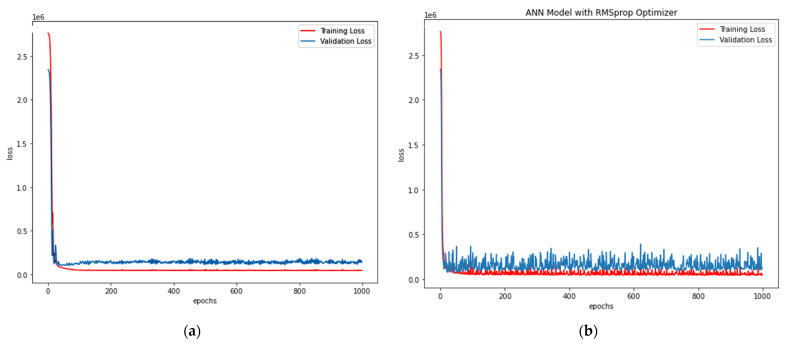
(**a**) Loss vs. Epochs of ANN-R Adam Optimisation in CFRP; (**b**) Loss vs. Epochs of ANN-RMSprop Optimisation in CFRP.

- Adam Optimiser in ANN-R: The estimated RMSE value is 380.81, where the R^2^ score is 0.16, and the *P_u_* (kN) values of the predicted loss with respect to the values and original values are plotted in Figure 11a.
- RMS prop Optimiser in ANN-R: estimated RMSE value is 359.93 where R^2^ score is 0.25. The *P_u_* (kN) values of the predicted loss with respect to the original values are plotted in Figure 11b.

The predicted values (blue dots) and the test values (red dots) of Figure 10 are nearer to each other, denoting that the ANN-R in CFRP model is a good fit.

From the above plots (Figure 11a,b), it can be seen that the ANN regressor with a RMSprop optimiser performs better than the ANN regressor with an Adam optimiser.

### 6.3. GFRP Dataset

**Figure 12:**
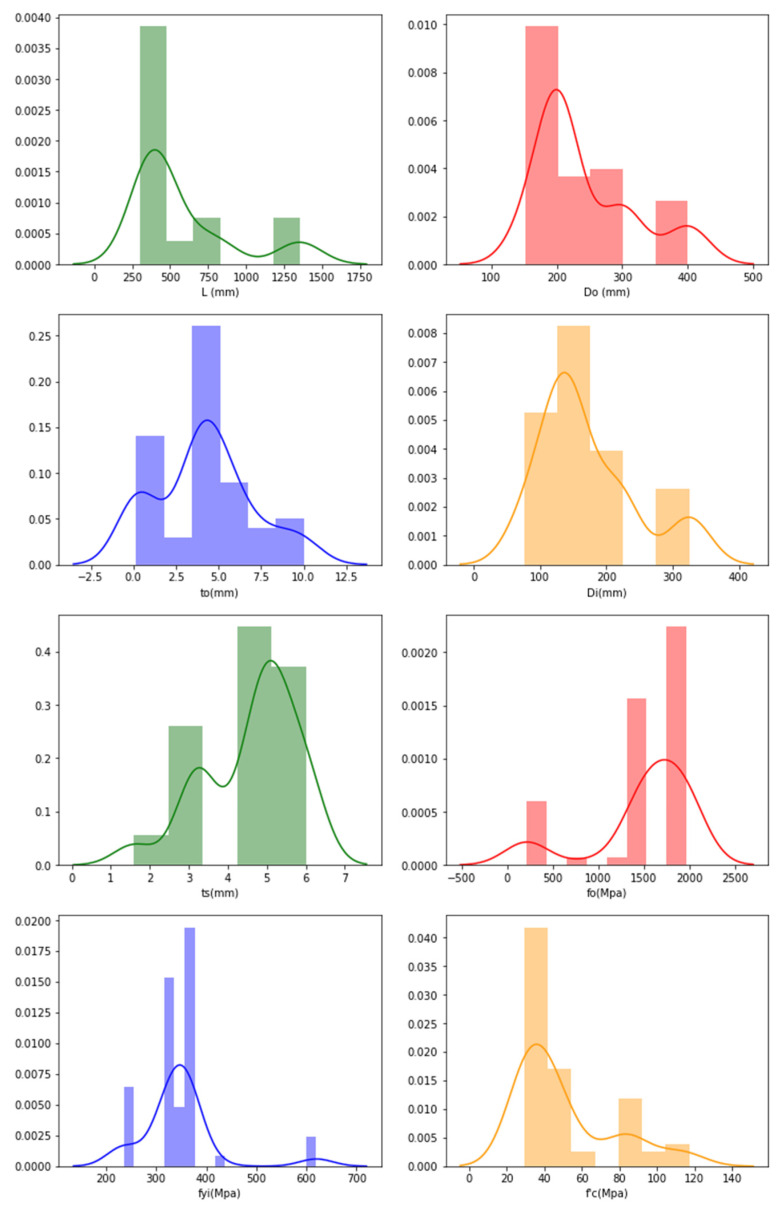
Frequency distribution in CFDST columns of GFRP representing features.

The frequency distributions of the features are plotted with the aim of verifying their distributions and the following plots are obtained through graphical representation as depicted in Figure 12 for GFRP datasets.

For the GFRP frequency distribution, it is understood from Figure 12 that, the data are distributed evenly and the density of the frequency decreases as the data increases. The frequency distribution is high for *L_o_* (300–350 mm), *D_o_* (150–300 mm_),_ *t_o_* (0.5–5 mm), *D_i_* (80–210 mm), *t_s_* (4–6 mm), *f_o_* (1500–2000 Mpa), *f_yi_* (300–400 Mpa) and f′_c_ (30–60 Mpa).

Predictions through regressor techniques:

(a) RFR: The estimated RMSE value is 670.26, where the R^2^ score is of 0.25;
(b) XGBR: The estimated RMSE is 569.86, where the R^2^ score is of 0.45;
(c) ABR: The estimated RMSE value is 549.96, where the R^2^ score is of 0.49;
(d) LR: The estimated RMSE value is 630.14, where the R^2^ score is of 0.33;
(e) RR: The estimated RMSE value is 585.16, where the R^2^ score is of 0.42.

Among the five regressor techniques above, the ABR could be inferred as a good fit for GFRP in the CFDST columns.

**Figure 13:**
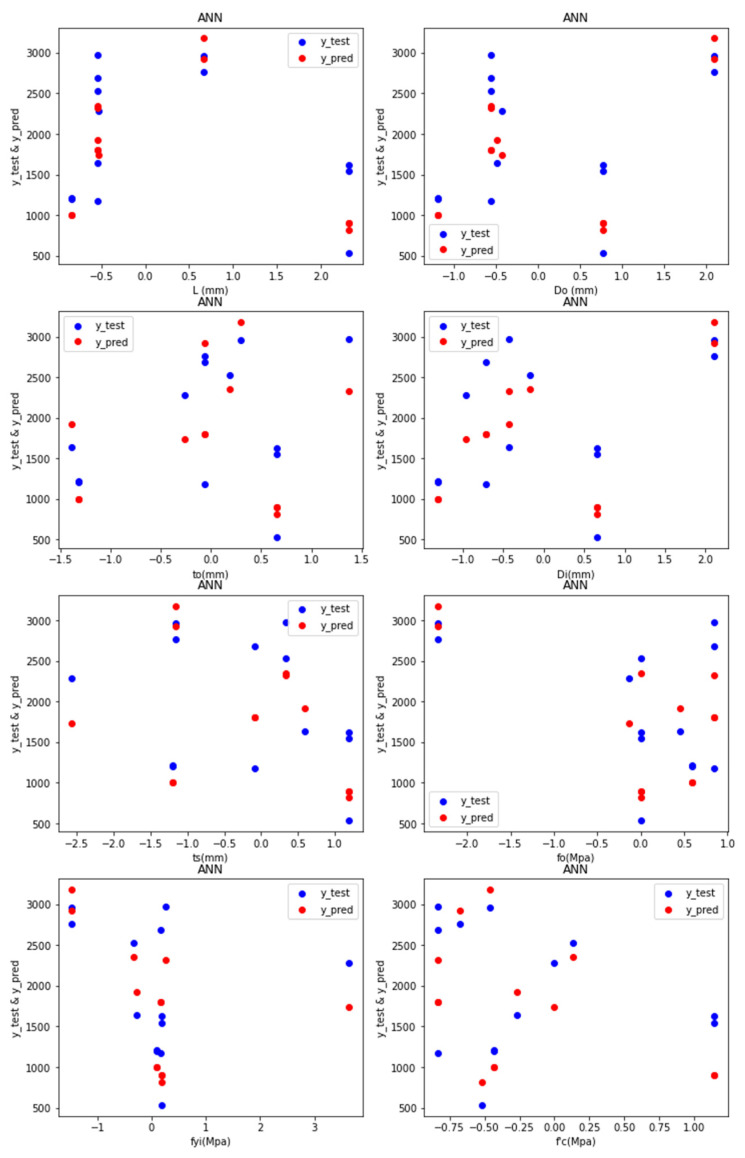
Predictions of ANN-R in GFRP.

(f) ANN-R:

Predictions: Figure 13 depicts the test values and predicted values of the ANN-R in GFRP, with the blue dots representing the predicted values and the red dots representing the test values:

The predicted values (blue dots) and the test values (red dots) from Figure 13 are nearer to each other, denoting that, the ANN-R in GFRP model is a good fit.

**Figure 14:**
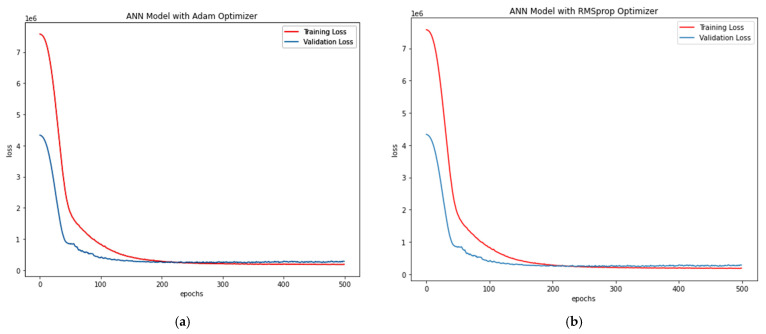
(**a**) Loss vs. Epochs of ANN-R Adam Optimisation in GFRP; (**b**) Loss vs. Epochs of ANN-R RMSprop Optimisation in GFRP.

- Adam Optimiser in ANN-R: estimated RMSE value is 493.80 where R^2^ score is 0.59, and the *Pu* (kN) values of the predicted loss with respect to values and original values are plotted in Figure 14a.
- RMS prop Optimiser in ANN-R: estimated RMSE value is 531.77 where R^2^ score is 0.52. The *Pu* (kN) values of the predicted loss with respect to and original values are plotted in Figure 14b.

From the above plots (Figure 14a,b) of ANN-R optimisation, it can be inferred that the ANN-R with an Adam optimiser performs better than the ANN regressor with a RMSprop optimiser.

### 6.4. PETFRP Dataset

**Figure 15:**
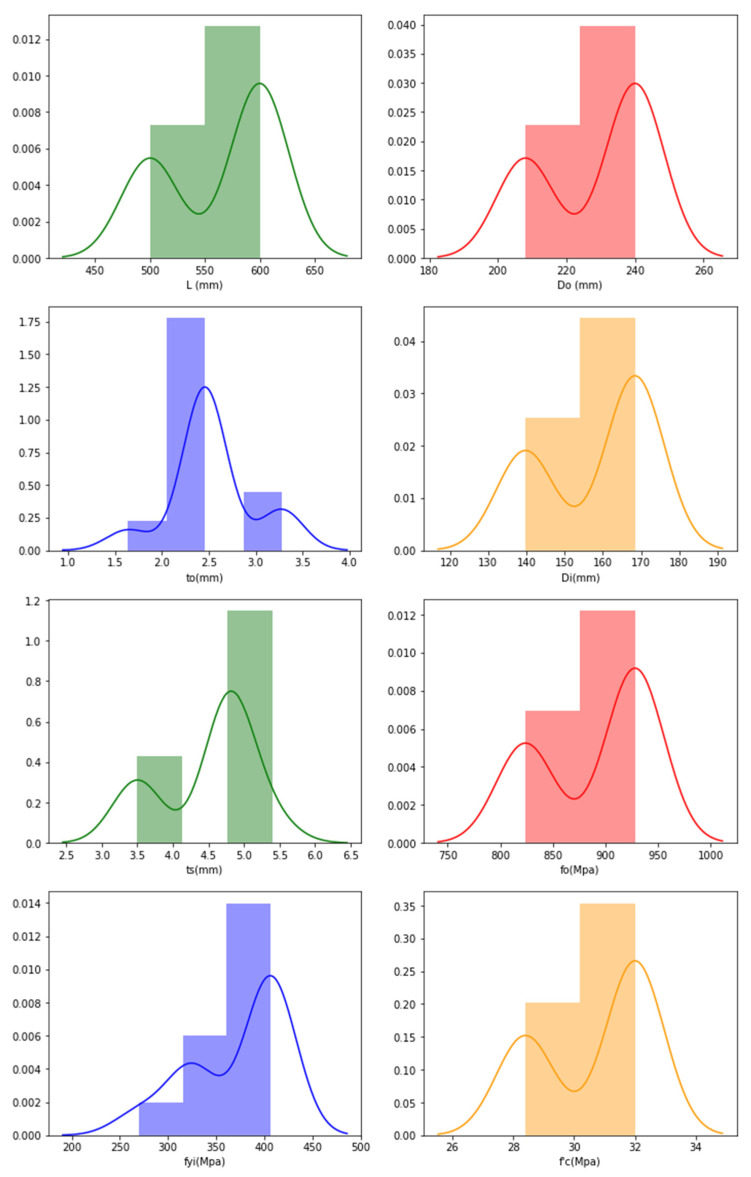
Frequency distribution in CFDST columns of PETFRP representing features.

The frequency distributions of the features are plotted with the aim of verifying their distributions, and the following plots are obtained through graphical representation as shown in Figure 15 for PETFRP datasets.

Predictions through regressor techniques:

(a) RFR: The estimated RMSE value is 204.39, where the R^2^ score is 0.71;
(b) XGBR: The estimated RMSE is 208.50, where the R^2^ score is 0.75;
(c) ABR: The estimated RMSE value is 206.84, where the R^2^ score is 0.70;
(d) LR: The estimated RMSE value is 200.63, where the R^2^ score is 0.72;
(e) RR: The estimated RMSE value is 200.58, where the R^2^ score is 0.72.

Among the five regressor techniques above, the RR could be inferred as a good fit for CFRP in the PETFRP columns.

**Figure 16:**
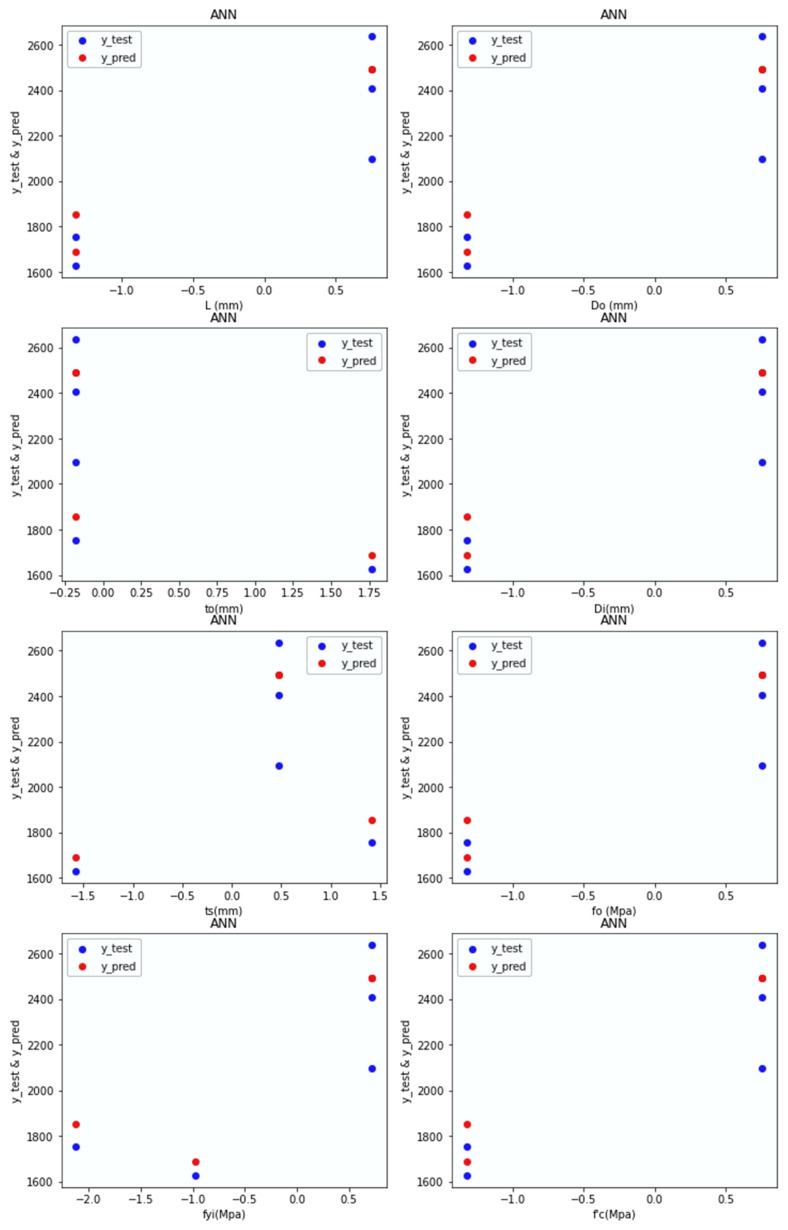
Predictions of ANN-R in PETFRP.

(f) ANN-R:

Predictions: Figure 16 depicts the test values and predicted values of the ANN-R in PETFRP, with the blue dots representing the predicted values and the red dots representing the test values:

For the PETFRP frequency distribution, it is understood from Figure 15 that the data are distributed evenly and, as the data increases, the density of the frequency decreases, too. The frequency distribution is high for *L_o_* (500–600 mm), *D_o_* (200–240 mm_),_ *t_o_* (1.5–2.5 mm), *D_i_* (140–170 mm), *t_s_* (4.5–5.5 mm), *f_o_* (800–95000 Mpa), *f_yi_* (250–400 Mpa), f′_c_ (25–30 Mpa).

The predicted values (blue dots) and the test values (red dots) from Figure 16 are nearer to each other, denoting that the ANN-R in the PETFRP model is a good fit.

**Figure 17:**
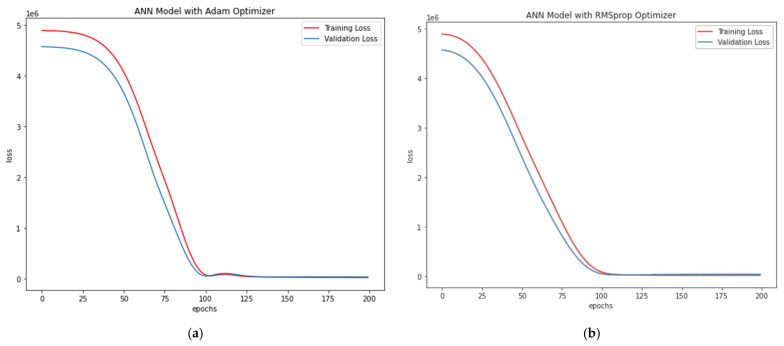
(**a**) Loss vs. Epochs of ANN-R Adam Optimisation in PETFRP; (**b**) Loss vs. Epochs of ANN-R RMSprop Optimisation in PETFRP.

- Adam Optimiser in ANN-R: The estimated RMSE value is 202.16, where the R^2^ score is 0.71, and the pu (kN) values of the predicted loss with respect to values and original values are plotted in Figure 17a.
- RMS prop Optimiser in ANN-R: The estimated RMSE value is 228.49, where the R^2^ score is 0.63. The pu (kN) values of the predicted loss with respect to and original values are plot-ted in Figure 17b.

From the above plots (Figure 17a,b) it could be witnessed that the ANN regressor with Adam optimiser performs better than the ANN regressor with RMSprop optimiser.

### 6.5. Steel Dataset

**Figure 18:**
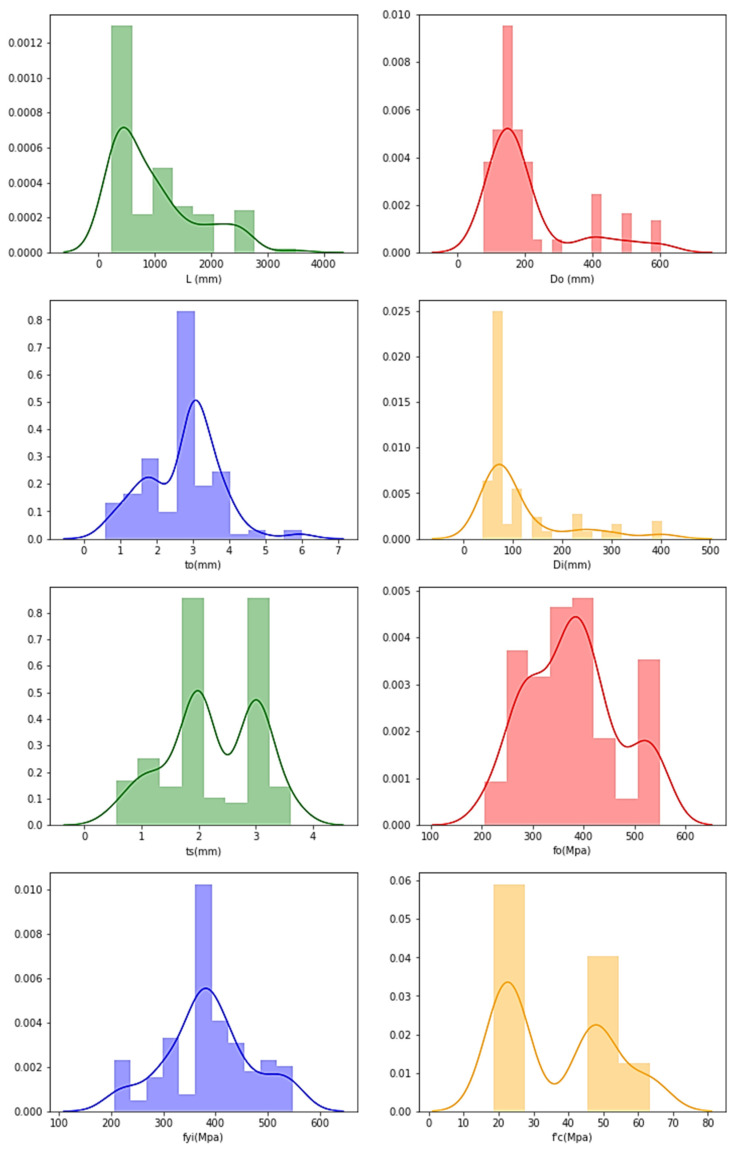
Frequency distribution in CFDST columns of STEEL representing features.

The fequency distributions of the features are plotted with the aim of verifying their distributions, and the following plots are obtained through graphical representation, as shown in Figure 18 for the Steel datasets.

Predictions through regressor techniques:

(a) RFR: The estimated RMSE value is 195.57, where the R^2^ score is 0.98;
(b) XGBR: The estimated RMSE is 202.54, where the R^2^ score is 0.97;
(c) ABR: The estimated RMSE value is 182.86, where the R^2^ score is 0.98;
(d) LR: The estimated RMSE value is 315.97, where the R^2^ score is 0.94;
(e) RR: The estimated RMSE value is 317.03, where the R^2^ score is 0.94.

Among the five regressor techniques above, the ABR could be inferred as a good fit for the Steel in CFDST columns.

**Figure 19:**
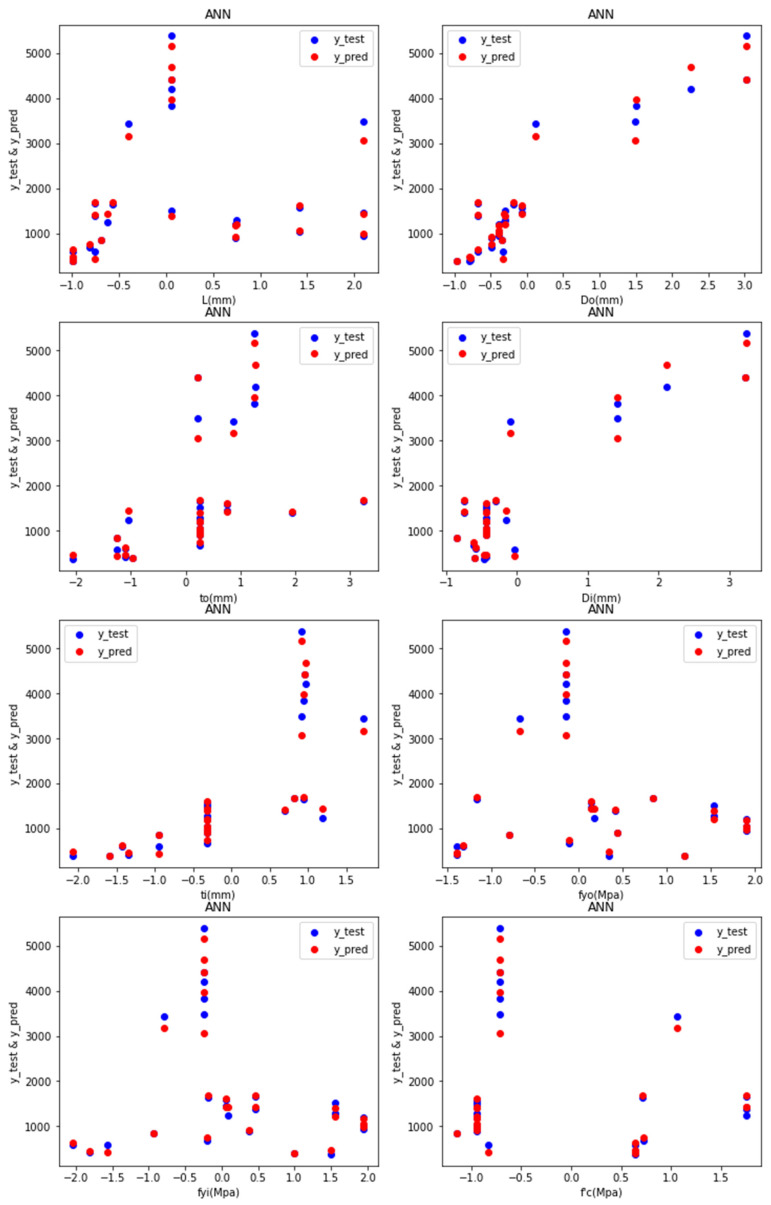
Predictions of ANN-R in STEEL.

(f) ANN-R:

Predictions: Figure 19 depicts the test values and predicted values of the ANN-R in AFRP, with the blue dots representing the predicted values and the red dots representing the test values:

For the STEEL frequency distribution, it is understood from Figure 18 that the data are distributed evenly and the density of the frequency decreases as the data increases. The frequency distribution is high for *L_o_* (300–500 mm), *D_o_* (150–250 mm_),_ *t_o_* (2–3 mm), *D_i_* (50–100 mm), *t_s_* (1.5–3.5 mm), *f_o_* (300–500 Mpa), *f_yi_* (350–400 Mpa) and f′_c_ (20–30 Mpa).

The predicted values (blue dots) and the test values (red dots) from Figure 19 are nearer to each other, denoting that the ANN-R in a STEEL model is a good fit.

**Figure 20:**
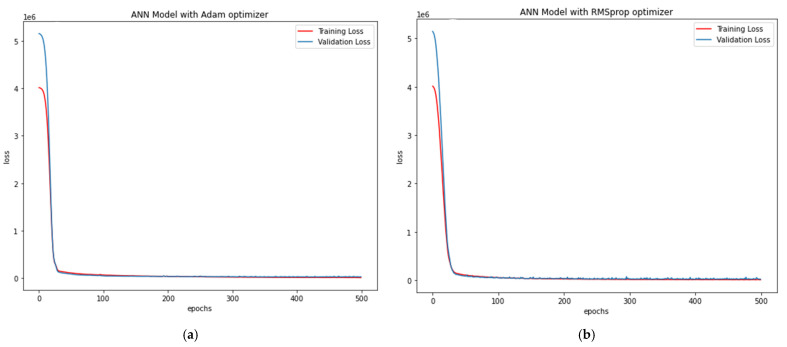
(**a**) Loss vs. Epochs of ANN-R Adam Optimisation in Steel; (**b**) Loss vs. Epochs of ANN-R RMSprop Optimisation in Steel.

- Adam Optimiser in ANN-R: The estimated RMSE value is 172.76, where the R^2^ score is 0.984, and the pu (kN) values of the predicted loss with respect to values and original values are plotted in Figure 20a.
- RMSprop Optimiser in ANN-R: The estimated RMSE value is 156.10, where the R^2^ score is 0.987. The pu (kN) values of the predicted loss with respect to and original values are plot-ted in Figure 20b.

From the above plots (Figure 20a,b) it can be witnessed that the ANN regressor with a RMSprop optimiser performs better than the ANN regressor with an Adam optimiser.

## 7. Development of the Predictive Equations

Based on the correlation and regression analysis, we have proposed two different equations depending on the hypothesis based on the variables *L*, *D_o_*, *t_o_*, *D_i_*, *t_S_*, *f_o_*, *f_yi_* and f′_c_. In this population, each solution consists of a randomly generated, unique combination of the coefficients, where all of the coefficients take values in (−1, +1). For each member of the population, the difference between the actual experimental axial load-carrying capacities and the p values computed by Equations (9) and (10) was calculated and stored in a vector with the length of the entire training set.

However, after a certain number of iterations, both the best and worst member coefficients nearly converged to the same values. Using the limit values of the best member coefficients, Equation (9) was obtained for DSTC with the outer FRP tube and the inner steel tube, and Equation (10) was obtained for DSTC with both inner and outer steel tubes.

(9) $$\;p=562+0.68\;L-2.58\;D_{o}+88.9\;t_{o}+4.27\;D_{i}+100.5\;t_{i}-0.140\;f_{o}+0.141\;f_{yi}+17.00\;f_{c}\;$$

(10) $$\;\;\;p=-1317-0.1109\;L+10.68\;D_{o}+236.4\;t_{o}-3.89\;D_{i}+143.4\;t_{i}+0.972\;f_{o}-0.880\;f_{yi}+10.26\;f_{c}$$

Finally, a list of all two equations proposed in this paper was formed. It should be noted that the proposed equations are data-driven, and the performance of those equations depends on the characteristics of the data used to develop the ML models. The equations are only applicable for the range of maximum and minimum values of the input parameters.

## 8. Performance Evaluation and Findings

Among the optimisers of the ANN regressor, Table 1 shows that:

- The ANN-R with an Adam optimiser is more effective than the RMSprop Optimiser with a RMSE score of 547.77 and R^2^ score of 0.57. Similarly, it can also be inferred that, among the existing empirical evaluation techniques and formulae aimed at estimating the ACC capacity of CFDST columns in AFRP dataset, the AdaBoost Regressor technique is the most effective, with a lower RMSE score (510.00) and higher R^2^ score (0.62).
- The ANN-R with a RMSprop Optimiser, more than the Adam optimiser, is effective with a RMSE score of 359.93 and R^2^ score of 0.25. Likewise, it can also be inferred that the Random Forest Regressor technique is the overall adopted empirical evaluation technique with the most successful formulae aimed at estimating the ACC capacity of CFDST columns in the CFRP dataset. The Random Forest Regressor technique is the most effective, with a lower RMSE score (355.50) and higher R^2^ score (0.27).
- The ANN-R with an Adam optimiser is more effective than the RMSprop Optimiser, with a RMSE score of 493.80 and R^2^ score of 0.59. Similarly, it could also be inferred that, among the overall adopted existing empirical evaluation techniques and formulae aimed at estimating the ACC capacity of CFDST columns in the GFRP dataset, the AdaBoost Regressor technique is the most effective, with a lower RMSE score (549.96) and a higher R^2^ score (0.49).
- The Adam Optimiser-based ANN-R is more effective than the RMSprop Optimiser, with a RMSE score of 202.16 and R^2^ score of 0.71. Correspondingly, it can also be inferred that the Ridge Regressor technique is the overall adopted empirical evaluation technique with the most successful formulae aimed at estimating the ACC capacity of the CFDST columns in the PETFRP dataset. The Ridge Regressor technique is the most effective, with a lower RMSE score (200.58) and higher R^2^ score (0.72).
- The ANN-R with a RMSprop Optimiser is more effective than the Adam optimiser, with a RMSE score of 156.10 and R^2^ score of 0.987. It can also be inferred that, among the overall adopted existing empirical evaluation techniques and formulae towards estimating the ACC capacity of CFDST columns in Steel dataset, the AdaBoost Regressor technique is the most effective, with a lower RMSE score (156.10) and higher R^2^ score (0.987).

Thus, it can be observed from the performance metrics that:

(a) For the Aramid FRP dataset, the ABR is effective, where the RMSE is 510.00, and the R^2^ is 0.62;
(b) For the Carbon FRP dataset, the RFR is effective, where the RMSE is 355.50, and the R^2^ is 0.27;
(c) For the Glass FRP dataset the ABR is effective, where the RMSE is 549.96, and the R^2^ is 0.49;
(d) For the Poly Ethylene Terephthalate FRP dataset the RR is effective, where the RMSE is 200.58, and the R^2^ is 0.72;
(e) For the Steel dataset the ABR is effective, where the RMSE is 182.26, and the R^2^ is 0.98.

## 9. Conclusions

This study focused on estimating and evaluating the ACC of the CFDST columns by examining the existing empirical formulae and utilising the parameters as the current study’s model’s features. The study adopted six regressors as the evaluation techniques, where AdaBoost Regression (ABR), Ridge Regression (RR), Lasso Regression (LR), ANN- Regression (ANN-R), XGBoost Regression (XGBR) and Random Forest as Regression (RFR) are adopted for evaluating the ACC of the CFDST. The data was acquired for the research through real-time data acquisition. The AFRP dataset contains 38 specimens, the CFRP dataset contains 59, the GFRP dataset contains 61, the PETFRP dataset contains 22 and the steel dataset contains 125. According to the study by Liao et al. (2021) [34], the sample sise has been determined for the current research

- The main perspective of this study is to indicate the applicability of the ANN technique to derive an effective statistical model for estimating the ultimate axial strength of CFDST composite columns. Moreover, the prediction performances of the design models generated from these techniques are shown statistically.
- The research developed has been used to estimate the axial compression capacity of the concrete-filled double-skin tubular columns with metallic and non-metallic composite materials, which is intended to be used in validating the better formula with statistical analysis. Through the evaluation outcomes, it was found that the ABR along with the RFR techniques in CFDST were reasonably more effective than the other techniques, and thus, it could be concluded that AdaBoost and Random-Forest Regressions are the effective empirical formulae to evaluate the ACC in CFDST columns through the Artificial Neural Network system.
- It can be concluded from the evaluation outcomes that for the outer skin and inner skin with ‘steel’ as the tube’s confinement, capacity would be higher where the R^2^ score is more than 0.90. Thus, Steel is more effective in the construction of CFDST columns than FRP-based CFDST columns. The developed computational model is valid and reliable, since the outcome yielded had neither a negative value nor a zero value.
- The same techniques and developed architecture could be compared and weighed against other CFDST column-based studies in the future. This research thus provides an effective base for future CFDST-oriented studies. Moreover, it provides the best model to adopt in estimating the ACC of CFDST columns in engineering out of a huge set of existing novel/empirical models. Thus, the study also shows that, given higher R^2^ scores and lower RMSE scores of the evaluation techniques proves, the adopted empirical formulae is quite effective, where it can be employed in similar research in other fields (medicine, management, etc.) to test the reliability, accuracy and validity of the variables prior to applying the test upon factors to derive outcomes.
- The availability of closed-form equations for accurate predictions of structural responses is beneficial in engineering practice. However, it should be noted that the developed equations are based on an experimental database consisting of 244 samples, and further studies in this area using larger databases are warranted. Furthermore, it should be noted that the results predicted by the developed equations are only valid within the range of the database used. In addition to experimental research, well-calibrated finite-element models could be used to improve the databases. Future study in this area might concentrate on predicting the axial load-carrying capacity under eccentric axial loading in addition to expanding the size of the database utilised in model training.

## Figures and Tables

**Table 1:** Performance Evaluation CFDST.

		RFR	XGBR	ABR	LR	RR	ANN R (Adam)	ANN R (RMSprop)
**AFRP**	**RMSE**	537.12	542.54	**510.00**	660.82	654.41	**547.77**	558.12
	**R^2^**	0.58	0.70	**0.62**	0.37	0.38	**0.57**	0.55
**CFRP**	**RMSE**	**355.50**	355.22	371.04	380.38	380.71	380.81	**359.93**
	**R^2^**	**0.27**	0.27	0.20	0.16	0.16	0.16	**0.25**
**GFRP**	**RMSE**	670.26	569.86	**549.96**	630.14	585.16	**493.80**	531.77
	**R^2^**	0.25	0.45	**0.49**	0.33	0.42	**0.59**	0.52
**PETFRP**	**RMSE**	204.39	208.50	206.84	355.50	**200.58**	**202.16**	228.49
	**R^2^**	0.71	0.70	0.70	0.27	**0.72**	**0.71**	0.63
**STEEL**	**RMSE**	195.57	202.54	**182.86**	315.97	317.03	172.76	**156.10**
	**R^2^**	0.98	0.97	**0.98**	0.94	0.94	0.984	**0.987**

## Data Availability

The datasets generated during and/or analysed during the current study are available from the corresponding author on reasonable request.

## Appendix A

**Table A1:** Experimental Dataset.

Specimens	*L* (mm)	*D_o_* (mm)	*t_o_* (mm)	*D_i_* (mm)	*t_s_* (mm)	*f_o_* (Mpa)	*f_yi_* (Mpa)	f′c (Mpa)	*P_u_* (kN)	Author	Year
**CFRP SPECIMENS (outer-CFRP, inner-steel)**											
DSTC-1	305	152.5	0.702	88.9	3.2	3800	314.2	113.8	1624	Fanggi and Ozbakkaloglu [35]	2013
DSTC-2	305	152.5	0.702	88.9	3.2	3800	314.2	113.8	1622		
DSTC-1	300	150	0.234	101.6	3.2	3626	302	37	914	Ozbakkaloglu and Fanggi [21]	2014
DSTC-2	300	150	0.234	101.6	3.2	3626	302	37	955		
DSTC-3	300	150	0.234	101.6	3.2	3626	302	36.7	1145		
DSTC-4	300	150	0.234	101.6	3.2	3626	302	36.7	1322		
DSTC-5	300	150	0.234	76.1	3.2	3626	358	36.9	911		
DSTC-6	300	150	0.234	76.1	3.2	3626	358	36.9	932		
DSTC-7	300	150	0.234	76.1	3.2	3626	351	36.4	1009		
DSTC-8	300	150	0.234	76.1	3.2	3626	351	36.4	1048		
DSTC-9	300	150	0.702	101.6	3.2	3626	302	37	1448		
DSTC-10	300	150	0.702	101.6	3.2	3626	302	37	1497		
DSTC-11	300	150	0.702	101.6	3.2	3626	302	106	1513		
DSTC-12	300	150	0.702	101.6	3.2	3626	302	106	1349		
DSTC-13	300	150	0.702	101.6	3.2	3626	302	106	2534		
DSTC-14	300	150	0.702	101.6	3.2	3626	302	106	3185		
DSTC-15	300	150	0.702	76.1	3.2	3626	358	106	2066		
DSTC-16	300	150	0.702	76.1	3.2	3626	358	106	1912		
DSTC-17	300	150	0.702	76.1	3.2	3626	351	107	2627		
DSTC-18	300	150	0.702	76.1	3.2	3626	351	107	2521		
DSTC-19	300	150	0.702	38.1	3.2	3626	411	106	2203		
DSTC-20	300	150	0.702	38.1	3.2	3626	411	106	2142		
DSTC-21	300	150	0.702	38.1	1.6	3626	434	106	2294		
DSTC-22	300	150	0.702	38.1	1.6	3626	434	106	2384		
DSTC-23	300	150	0.702	38.1	1.6	3626	1360	108	2175		
DSTC-24	300	150	0.702	38.1	1.6	3626	1414	108	2533		
DN2-60-I	300	150	0.334	60	4	3400	394	32	1490	Qi Cao [35]	2017
DN2-60-II	300	150	0.334	60	4	3400	394	32	1560		
DE2-60-I	300	150	0.334	60	4	3400	394	26	1255		
DE2-60-II	300	150	0.334	60	4	3400	394	26	1362		
DN1-89-I	300	150	0.167	89	4	3400	391	32	1118		
DN1-89-II	300	150	0.167	89	4	3400	391	32	1103		
DE1-89-I	300	150	0.167	89	4	3400	391	26	994		
DE1-89-II	300	150	0.167	89	4	3400	391	26	1001		
DN2-89-I	300	150	0.334	89	4	3400	391	32	1438		
DN2-89-II	300	150	0.334	89	4	3400	391	32	1373		
DE2-89-I	300	150	0.334	89	4	3400	391	26	1341		
DE2-89-II	300	150	0.334	89	4	3400	391	26	1365		
DN2-114-I	300	150	0.334	114	4.5	3400	332	32	1340		
DN2-114-II	300	150	0.334	114	4.5	3400	332	32	1313		
DE2-114-I	300	150	0.334	114	4.5	3400	332	26	1349		
DE2-114-II	300	150	0.334	114	4.5	3400	332	26	1342		
DN2-114N-I	300	150	0.334	114	4.5	3400	332	32	1767		
DN2-114N-II	300	150	0.334	114	4.5	3400	332	32	1724		
DE2-114N-I	300	150	0.334	114	4.5	3400	332	26	1945		
DE2-114N-II	300	150	0.334	114	4.5	3400	332	26	1953		
DC28(1)	300	153	1.5	42.2	2	3400	289	39.8	1133	Ying Wu Zhou [36]	2017
DC28(2)	300	153	1.5	42.2	2	3400	289	39.8	1118		
DC28(3)	300	153	1.5	42.2	2	3400	289	39.8	1113		
DC36(1)	300	153	1.5	54.7	2	3400	366	39.8	1082		
DC36(2)	300	153	1.5	54.7	2	3400	366	39.8	1078		
DC36(3)	300	153	1.5	54.7	2	3400	366	39.8	1060		
DC47(1)	300	153	1.5	70.8	2	3400	288	39.8	1026		
DC47(2)	300	153	1.5	70.8	2	3400	288	39.8	971		
DC47(3)	300	153	1.5	70.8	2	3400	288	39.8	956		
CCFST-1	500	165	0.167	164.8	2	2878	275	31.2	1948.8	Jun Deng [37]	2017
CCFST-2	500	165	0.167	164.8	2	2878	275	31.2	1948.8		
CCFST-3	500	165	0.167	164.8	2	2878	275	31.2	1948.8		
CCFST-4	500	165	0.167	164.8	2	2878	275	31.2	1948.8		
**AFRP SPECIMENS (outer-AFRP, inner-steel)**											
DSTC-3	305	152.5	0.8	88.9	3.2	2900	314.2	113.8	1919	Fanggi and Ozbakkaloglu [20]	2013
DSTC-4	305	152.5	0.8	88.9	3.2	2900	314.2	113.8	1965		
DSTC-5	305	152.5	1.2	88.9	3.2	2900	314.2	113.8	2247		
DSTC-6	305	152.5	1.2	88.9	3.2	2900	314.2	113.8	2251		
DSTC-7	305	152.5	0.6	88.9	3.2	2900	314.2	49.8	1664		
DSTC-8	305	152.5	0.6	88.9	3.2	2900	314.2	49.8	1567		
DSTC-9	305	152.5	1.2	60.3	3.6	2900	459.4	113.8	2745		
DSTC-10	305	152.5	1.2	60.3	3.6	2900	459.4	113.8	2783		
DSTC-11	305	152.5	1.2	88.9	5.5	2900	407.7	113.8	2843		
DSTC-12	305	152.5	1.2	88.9	5.5	2900	407.7	113.8	2846		
DSTC-13	305	152.5	1.2	114.3	6.02	2900	342.3	113.8	2331		
DSTC-14	305	152.5	1.2	114.3	6.02	2900	342.3	113.8	2228		
DSTC-15	305	152.5	1.2	89	3.5	2900	461.8	113.8	1482		
DSTC-16	305	152.5	1.2	89	3.5	2900	461.8	113.8	1346		
DSTC-1	305	152.5	0.6	88.9	3.2	2663	320	47.3	2261	Togay Ozbakkaloglu [38]	2015
DSTC-2	305	152.5	0.6	88.9	3.2	2663	320	47.3	2217		
DSTC-3	305	152.5	1.2	88.9	3.2	2663	320	75.95	3844		
DSTC-4	305	152.5	1.2	88.9	3.2	2663	320	75.95	3789		
DSTC-5	305	152.5	1.2	88.9	3.2	2663	320	104.6	3534		
DSTC-6	305	152.5	1.2	88.9	3.2	2663	320	104.6	3357		
DSTC-7	305	152.5	1.2	88.9	5.5	2663	408	104.6	3713		
DSTC-8	305	152.5	1.2	88.9	5.5	2663	408	104.6	4110		
DSTC-9	305	152.5	1.2	60.3	3.6	2663	319	104.6	3496		
DSTC-10	305	152.5	1.2	60.3	3.6	2663	319	104.6	3314		
DSTC-11	305	152.5	1.2	101.6	3.2	2663	310	104.6	3816		
DSTC-12	305	152.5	1.2	101.6	3.2	2663	310	104.6	3678		
DSTC-13	305	152.5	1.2	114.3	6.02	2663	449	104.6	4293		
DSTC-14	305	152.5	1.2	114.3	6.02	2663	449	104.6	3964		
DSTC-15	305	152.5	1.2	101.6	3.2	2663	310	104.6	2022		
DSTC-16	305	152.5	1.2	101.6	3.2	2663	310	104.6	2021		
DSTC-17	305	152.5	1.2	101.6	3.2	2663	310	104.6	2011		
DSTC-18	305	152.5	1.2	101.6	3.2	2663	310	104.6	1839		
DSTC-1C	305	152.5	0.6	88.9	3.2	2390	320	42.5	2072		
DSTC-2C	305	152.5	0.6	88.9	3.2	2390	320	42.5	1936		
DSTC-3C	305	152.5	1.2	88.9	3.2	2390	320	82.4	3679		
DSTC-4C	305	152.5	1.2	88.9	3.2	2390	320	82.4	3812		
DSTC-5C	305	152.5	1.2	60.3	3.6	2390	319	82.4	3515		
DSTC-6C	305	152.5	1.2	60.3	3.6	2390	319	82.4	3632		
**GFRP SPECIMENS (outer-GFRP, inner-steel)**											
DS1A	305	152.5	0.17	76.1	3.2	1825.5	352.7	39.6	793.75	Teng [18]	2007
DS2A	305	152.5	0.34	76.1	3.2	1825.5	352.7	39.6	1044.2		
DS3A	305	152.5	0.51	76.1	3.2	1825.5	352.7	39.6	1214		
DS1B	305	152.5	0.17	76.1	3.2	1825.5	352.7	39.6	829.27		
DS2B	305	152.5	0.34	76.1	3.2	1825.5	352.7	39.6	1024.8		
DS3B	305	152.5	0.51	76.1	3.2	1825.5	352.7	39.6	1201.9		
FS-Y0-C30-T4	800	400	4	325	3.25	215	235	29.9	2824	Zhe Xiong [39]	2018
FS-Y25-C30-T4	800	400	4	325	3.25	215	235	32.2	2714		
FS-Y50-C30-T4	800	400	4	325	3.25	215	235	30.7	2884		
FS-Y75-C30-T4	800	400	4	325	3.25	215	235	32.2	2694		
FS-Y100-C30-T4	800	400	4	325	3.25	215	235	33.4	2765		
FS-Y100-C40-T4	800	400	4	325	3.25	215	235	33.4	2860		
FS-Y100-C30-T4.5	800	400	4.5	325	3.25	215	235	33.4	3314		
FS-Y100-C30-T5	800	400	5	325	3.25	215	235	38.9	2962		
D30-A4-F80-M1	400	200	4	140	5	1970	365.8	29.3	2563.7	Bing Zhang [40]	2020
D30-A4-F80-M2	400	200	4	140	5	1970	365.8	29.3	2359.4		
D30-B4-F80-M1	400	200	4	120	4.5	1970	358.7	29.3	2685.5		
D30-B4-F80-M2	400	200	4	120	4.5	1970	358.7	29.3	2735.5		
D30-A4-F60-M1	400	200	4	140	5	1970	365.8	29.3	2186.6		
D30-A4-F60-M2	400	200	4	140	5	1970	365.8	29.3	2185.5		
D30-B4-F60-M1	400	200	4	120	4.5	1970	358.7	29.3	2156		
D30-B4-F60-M2	400	200	4	120	4.5	1970	358.7	29.3	2109.2		
D30-A8-F60-M1	400	200	8	140	5	1970	365.8	29.3	3100.6		
D30-A8-F60-M2	400	200	8	140	5	1970	365.8	29.3	2975		
D30-A4-F45-M1	400	200	4	140	5	1970	365.8	29.3	1207.3		
D30-A4-F45-M2	400	200	4	140	5	1970	365.8	29.3	1232.3		
D30-B4-F45-M1	400	200	4	120	4.5	1970	358.7	29.3	1284.9		
D30-B4-F45-M2	400	200	4	120	4.5	1970	358.7	29.3	1176.6		
D30-A8-F45-M1	400	200	8	140	5	1970	365.8	29.3	1562.7		
D30-A8-F45-M2	400	200	8	140	5	1970	365.8	29.3	1441.2		
M1	400	205.3	0.17	140.3	5.3	1752	325.5	43.9	1824	Yu and Zhang [41]	2012
M2	400	205.3	0.34	140.3	5.3	1752	325.5	43.9	1799		
F1	400	205.3	0.17	140.3	5.3	1752	325.5	43.9	1798		
F2	400	205.3	0.34	140.3	5.3	1752	325.5	43.9	1724		
PU1	400	205.3	0.17	140.3	5.3	1752	325.5	43.9	1783		
PU2	400	205.3	0.34	140.3	5.3	1752	325.5	43.9	1794		
PR1	400	205.3	0.17	140.3	5.3	1752	325.5	43.9	1774		
PR2	400	205.3	0.34	140.3	5.3	1752	325.5	43.9	1637		
F4-24-E325	2032	610	9.5	356	6.4	575	324	35.6	-	Omar [42]	2017
F4-24-E344	2032	610	9.5	406	12.7	575	324	39.8	-		
F4-24-P124-R	2032	610	3.2	406	6.4	575	324	39.8	-		
D37-6-0.2	1350	300	6	219	6	-	360.3	37.4	530.8	Zhang [43]	2015
D56-6-0.2	1350	300	6	219	6	-	360.3	56	668.3		
D80-6-0.4	1350	300	6	219	6	-	360.3	80	1546		
D80-6-0.4-S	1350	300	6	219	6	-	360.3	80	1624		
D80-10-0.4	1350	300	10	219	6	-	360.3	82.7	1479		
D116-6-0.2	1350	300	6	219	6	-	360.3	116.4	1060		
D116-6-0.4	1350	300	6	219	6	-	360.3	117.3	2116		
D116-10-0.4	1350	300	10	219	6	-	360.3	114.8	2103		
D54-2FW-M	400	200	2.2	159	5	-	320.4	54.1	1965	Zhang [22]	2017
D54-4FW-M	400	200	4.7	159	5	-	320.4	54.1	2530		
D84-4FW-M1	400	200	4.7	159	5	-	320.4	84.6	4461		
D84-4FW-M2	400	200	4.7	159	5	-	320.4	84.6	2650		
D84-4FW-MB	400	200	4.7	120	4.5	-	419.5	84.6	2763		
D84-9FW-M	400	200	9.5	159	5	-	320.4	84.6	3413		
D104-4FW-M	400	200	4.7	159	5	-	320.4	104.6	2616		
D104-9FW-M	400	200	9.5	159	5	-	320.4	104.6	3512		
D40-6FW-M	600	300	6	219	6	-	319.4	40.9	6002		
D66-6FW-M	600	300	6	219	6	-	319.4	66.1	5284		
D85-6FW-M	600	300	6	219	6	-	319.4	85.8	5482		
D85-10FW-M	600	300	10	219	6	-	319.4	85.8	7089		
**PETFRP SPECIMENS (outer-PETFRP, inner-steel)**											
DSTC-A2-I	500	208	1.638	139.7	3.5	823.9	325	28.4	1268	Tao Yu [44]	2017
DSTC-A2-II	500	208	1.638	139.7	3.5	823.9	325	28.4	1305		
DSTC-A3-I	500	208	2.457	139.7	3.5	823.9	325	28.4	1424		
DSTC-A3-II	500	208	2.457	139.7	3.5	823.9	325	28.4	1581		
DSTC-A4-I	500	208	3.276	139.7	3.5	823.9	325	28.4	1658		
DSTC-A4-II	500	208	3.276	139.7	3.5	823.9	325	28.4	1627		
DSTC-B3-I	500	208	2.457	139.7	5.4	823.9	270	28.4	1755		
DSTC-B3-II	500	208	2.457	139.7	5.4	823.9	270	28.4	1897		
**STEEL SPECIMENS (outer-steel, inner-steel)**											
A2-l	230	75.4	1.29	62.7	1.23	486	470	46.2	348	Wei et al. [45]	1995
A2-2	230	75.2	1.19	62.4	1.2	486	470	46.2	348		
A3-l	230	76.3	1.78	62	1	486	470	46.2	395		
A3-2	230	76.3	1.74	62	0.94	512	470	46.2	395		
B2-1	230	81.5	1.11	62.7	1.14	524	470	46.2	386		
B2-2	230	81.5	1.14	62.2	1.13	524	470	46.2	395		
Cl-I	230	87.4	0.99	61.8	0.87	428	452	46.2	378		
Cl-2	230	87.3	0.94	61.6	0.88	428	452	46.2	385		
D I-I	230	99.7	0.59	80.3	0.55	409	474	46.2	283		
D4-1	230	99.9	0.7	74	0.62	409	512	46.2	380		
D5-1	230	99.8	0.66	61.4	0.55	409	432	46.2	443		
D6-1	230	101.7	1.61	61.5	0.56	409	432	46.2	644		
E2- l	230	101.4	1.56	63.4	1.15	255	216	46.2	477		
E3- I	230	101.5	1.65	76.1	1.19	255	235	46.2	417		
E4-I	230	114.3	1.64	63.5	1.12	262	216	46.2	598		
E5-I	230	114.3	1.64	76.1	1.14	262	235	46.2	551		
E6-I	230	114.3	1.64	88.9	1.56	262	286	46.2	524		
cc2a	540	180	3	48	3	275.9	396.1	47.4	1790	Tao et al. [9]	2004
cc2b	540	180	3	48	3	275.9	396.1	47.4	1791		
cc3a	540	180	3	88	3	275.9	370.2	47.4	1648		
cc3b	540	180	3	88	3	275.9	370.2	47.4	1650		
cc4a	540	180	3	140	3	275.9	342	47.4	1435		
cc4b	540	180	3	140	3	275.9	342	47.4	1358		
cc5a	342	114	3	58	3	294.5	374.5	47.4	904		
cc5b	342	114	3	58	3	294.5	374.5	47.4	898		
cc6a	720	240	3	114	3	275.9	294.5	47.4	2421		
cc6b	720	240	3	114	3	275.9	294.5	47.4	2460		
cc7a	900	300	3	165	3	275.9	320.5	47.4	3331		
cc7b	900	300	3	165	3	275.9	320.5	47.4	3266		
O1I1	400	114.3	6	48.3	2.9	454	425	63.4	1665	Zhao et al. [46]	2010
O2I1	400	114.3	4.8	48.3	2.9	416	425	63.4	1441		
O3I1	400	114.3	3.6	48.3	2.9	453	425	63.4	1243		
O4I1	400	114.3	3.2	48.3	2.9	430	425	63.4	1145		
O5I2	400	165.1	3.5	101.6	3.3	433	394	63.4	1629		
O6I2	500	165.1	3	101.6	3.2	395	394	63.4	1613		
O7I2	500	163.8	2.35	101.6	3.2	395	394	63.4	1487		
O8I2	500	163	1.95	101.6	3.2	395	394	63.4	1328		
O9I2	500	162.5	1.7	101.6	3.2	395	394	63.4	1236		
c10-375	450	158	0.9	38	0.9	221	221	18.7	635	Uenaka et al. [33]	2010
c10-750	450	159	0.9	76	0.9	221	221	18.7	540		
c16-375	450	158	1.5	39	1.5	308	308	18.7	851.6		
c16-750	450	158	1.5	77	1.5	308	308	18.7	728.1		
c16-1125	450	158	1.5	114	1.5	308	308	18.7	589		
c23-375	450	158	2.14	40	2.14	286	286	18.7	968.2		
c23-750	450	158	2.14	77	2.14	286	286	18.7	879.1		
c23-1125	450	157	2.14	115	2.14	286	286	18.7	703.6		
DC-1	1324	120	1.96	60	1.96	311	380	53.45	779.1	Han et al. [47]	2011
DC-2	1324	120	1.96	60	1.96	311	380	53.45	836.9		
DCc-1	1324	120	1.96	60	1.96	311	380	53.45	789.9		
DCc-2	1324	120	1.96	60	1.96	311	380	53.45	715.4		
1	660	220	3.62	159	3.62	319.6	319.6	52.7	2537	Han et al. [48]	2011
2	660	220	3.62	159	3.62	319.6	319.6	52.7	2566		
3	660	220	3.62	106	3.62	319.6	319.6	52.7	3436		
4	660	220	3.62	106	3.62	319.6	319.6	52.7	3506		
7	660	220	3.62	159	3.62	319.6	319.6	52.7	2908		
8	660	220	3.62	159	3.62	319.6	319.6	52.7	2860		
DCS500-4-300A	998	500.2	4.02	301.6	3.02	366	366	25.32	4206	Chen et al. [49]	2015
DCS500-4-300B	1001	500.3	4.03	302.1	3.01	366	366	25.32	4606		
DCS500-4-300C	1000	500.1	4.01	300.9	3	366	366	25.32	4789		
DCS500-3-300A	1001	498.9	3.01	299.8	3	366	366	25.32	4162		
DCS500-3-300B	1002	498.5	2.99	302.1	2.98	366	366	25.32	3886.5		
DCS500-3-300C	1000	499.6	3.02	301.5	2.99	366	366	25.32	3882		
DCS600-4-400A	1001	601.1	4.01	401.2	2.98	366	366	25.32	5383.5		
DCS600-4-400B	1003	602.3	4.02	402.1	2.97	366	366	25.32	5370.5		
DCS600-4-400C	1001	603.4	3.98	401.5	3.02	366	366	25.32	4820		
DCS600-3-400A	999	601.5	2.98	399.8	3.01	366	366	25.32	4415		
DCS600-3-400B	999	601.2	3.02	400.1	3.02	366	366	25.32	4084.5		
CDCS400-3-1000	1002	400.2	3.01	241.2	3.01	366	366	25.32	3423		
CDCS400-3-2000	2003	400.6	3.02	240.5	3	366	366	25.32	3013		
CDCS400-3-2500	2501	398.2	3	239.8	3	366	366	25.32	3256.5		
CDCS400-3-3500	3502	398.6	3	239.6	3.02	366	366	25.32	2923		
DCS400-4-1000	1001	401.2	4	240.2	3	366	366	25.32	3828		
DCS400-4-2000B	2005	400.7	4.02	240.1	3.02	366	366	25.32	3542		
DCS400-4-2500	2502	400.3	4.05	240	3.05	366	366	25.32	3790		
DCS400-3-1000	1002	401.2	3.02	240	2.99	366	366	25.32	2990		
DCS400-3-2500	2498	400.1	2.98	240.2	2.98	366	366	25.32	3490		
S139.2-1.0	998	139.2	3	76	2	418	418	21.81	1059.2	Essopjee and Dundu [50]	2015
S139.2-1.0	1001	139.2	3	76	2	418	418	21.81	1056.1		
S139.2-1.5	1500	139.2	3	76	2	418	418	21.81	905.5		
S139.2-1.5	1503	139.2	3	76	2	418	418	21.81	901.6		
S139.2-2.0	2000	139.2	3	76	2	418	418	21.81	831.7		
S139.2-2.0	1998	139.2	3	76	2	418	418	21.81	837.4		
S139.2-2.5	2502	139.2	3	76	2	418	418	21.81	732.1		
S139.2-2.5	2498	139.2	3	76	2	418	418	21.81	729		
S152.4-1.0	1003	152.4	3	76	2	549	549	21.81	1263.5		
S152.4-1.0	1002	152.4	3	76	2	549	549	21.81	1254.9		
S152.4-1.5	1497	152.4	3	76	2	549	549	21.81	1195.6		
S152.4-1.5	1503	152.4	3	76	2	549	549	21.81	1191.2		
S152.4-2.0	1997	152.4	3	76	2	549	549	21.81	1047.3		
S152.4-2.0	2000	152.4	3	76	2	549	549	21.81	1041.6		
S152.4-2.5	2498	152.4	3	76	2	549	549	21.81	941.4		
S152.4-2.5	2500	152.4	3	76	2	549	549	21.81	949		
S165.1-1.0	998	165.1	3	76	2	516	516	21.81	1512.3		
S165.1-1.0	999	165.1	3	76	2	516	516	21.81	1510.6		
S165.1-1.5	1504	165.1	3	76	2	516	516	21.81	1286.4		
S165.1-1.5	1498	165.1	3	76	2	516	516	21.81	1275.1		
S165.1-2.0	2003	165.1	3	76	2	516	516	21.81	1187.2		
S165.1-2.0	1998	165.1	3	76	2	516	516	21.81	1199.8		
S165.1-2.5	2498	165.1	3	76	2	516	516	21.81	1028		
S165.1-2.5	2502	165.1	3	76	2	516	516	21.81	1036.5		
S193.7-1.0	1003	193.7	3.5	76	2	391	391	21.81	2010		
S193.7-1.0	1000	193.7	3.5	76	2	391	391	21.81	2030		
S193.7-1.5	1502	193.7	3.5	76	2	391	391	21.81	1730		
S193.7-1.5	1500	193.7	3.5	76	2	391	391	21.81	1720		
S193.7-2.0	1998	193.7	3.5	76	2	391	391	21.81	1581.6		
S193.7-2.0	2003	193.7	3.5	76	2	391	391	21.81	1584.1		
S193.7-2.5	2503	193.7	3.5	76	2	391	391	21.81	1451.4		
S193.7-2.5	2497	193.7	3.5	76	2	391	391	21.81	1458.7		
